# Field-based screening of selected oral antibiotics in Belize

**DOI:** 10.1371/journal.pone.0234814

**Published:** 2020-06-17

**Authors:** Danladi Chiroma Husaini, Uppala Jyana Venkata Kishan, Chen-Yi Wu, Elsbeth Nerissa Guerra, Cindy J. Bush, Ayleen Walewska Perez, Israel Coc

**Affiliations:** 1 Pharmacy Program, Faculty of Health Sciences, University of Belize, Belmopan City, Cayo District, Belize; 2 La Sante Pharmacy, San Ignacio Town, Cayo District, Belize; 3 Friendly Pharmacy, Belmopan City, Cayo District, Belize; 4 CJS Pharmacy, Benque Viejo del Carmen, Cayo District, Belize; 5 Genesis Medical Clinic, Orange Walk Town, Orange Walk District, Belize; 6 Codd’s Pharmacy, Belmopan City, Cayo District, Belize; College of Pharmacy & Health Sciences, UNITED STATES

## Abstract

**Introduction:**

The presence of poor quality antibiotics on the market has contributed to the antibiotics resistance and global threat to public health. Antibiotic resistance is now a global concern. One area to address this issue is by evaluating the quality of antibiotics accessible to the public. The purpose of this study was to test and compare (with corresponding pharmacopeia) the quality of common oral antibiotics available in the country of Belize with a view to providing base-line data on the testing of medications imported to the country for public consumption. The study focused only on level 2 field-based screening quality assurance on three Key Access Antibiotics from the World Health Organization (WHO) Model List of Essential Medicines.

**Methods:**

Five brands of antibiotic tablets/capsules with denoted pharmacopeia imported into the country of Belize were tested for quality at The University of Belize pharmacy laboratory. A sample of 30 tablets/capsules each of the selected antibiotic brand were used for study. Visual inspection and weight variation were done for each sample while Monsanto type tablet hardness tester, Roche^@^Tablet Friability Test Apparatus (single drum), and Ajanta^@^ Tablet Disintegration Test Apparatus (double basket) were conducted on selected antibiotics. Results were recorded and compared with corresponding pharmacopoeia references.

**Results:**

Most of the samples collected passed performed tests. Only a few samples from both BP and USP antibiotics failed in visual inspection and weight variation tests. All antibiotics tested conformed to their corresponding pharmacopeia reference in terms of friability and disintegration time.

**Conclusion:**

Most of the selected antibiotics passed performed tests when compared with their pharmacopeia. Only a few samples from both BP and USP antibiotics failed the tests conducted. There is need for regular quality assurance tests on all medications imported to Belize especially antibiotics.

## Introduction

The presence of poor quality antibiotics on the market has contributed in no small measure to the global antibiotics resistance and a threat to public health. Antibiotic resistance has become a global emergency that healthcare professionals are confronted with in recent years [1]. In the United States alone, at least 2 million individuals were reported to be affected by antibiotic resistant bacteria [2]. The Center for Disease Control (CDC) and prevention reported more than 23,000 deaths in the United States 2013 [3] and 33,000 deaths in Europe [4] as a result of antibiotic resistance. The threat of antibiotics resistance does not only apply to the western developed countries but also in developing countries where health care provision is a major challenge. In Thailand for instance, antibiotic resistance has accounted for more than 38,000 deaths [5]. Between 2000 and 2010 alone, a total increase of antibiotic consumption has been observed in 71 countries. This increase has skyrocketed up to 45% for last-resort antibiotics, whose usage is only reserved when other antibiotic treatments are no longer effective [6]. The increased consumption of antibiotics can only result in the increase risk for developing resistance.

While bacteria naturally developed resistance over time, many factors have been reported to accelerate this process. These factors include misuse and overuse of antibiotics, inappropriate prescribing, extensive agricultural usage and inadequate discovery of new antibiotics [7–12].

Furthermore, poor quality of antibiotics available to the public may lead to the development of antibiotics resistance. The quality of a medication is affected by low drug potency, poor formulation and/or presence of impurities [13, 14]. Although there is limited data that link the quality of specific antibiotics to resistance in particular diseases, evidence shows that resistance develops when bacteria is being exposed to sub-therapeutic doses of antibiotics. This will contribute to treatment regimens appearing as ineffective resulting in stronger antibiotics being needlessly introduced which will further escalate the possibilities of resistance [1].

Presently, the Belize Ministry of Health (MOH) through its drug regulatory department is actively implementing and enforcing the Belize Antibiotics Act to ensure compliance. The Antibiotics Act ensures that antibiotics are only accessed based on prescription from a licensed medical practitioner among other things. The incidence of poor quality antibiotics especially in developing countries like Belize pose a threat to public health leading to poor management and the development of antibiotic resistance. The issues of limited human and financial resources are an additional challenge which is also characterized in many developing countries. These challenges put huge strain in the number of personnel required for regulatory affairs and enforcements. Furthermore, lack of funding makes it difficult to conduct complex quality assurance tests required on medications especially antibiotics imported into the country. The apparent lack of public awareness on the quality of medications as well as the severity of global antibiotic resistance crisis makes monitoring antibiotics use in Belize a challenging task for the MOH. This study therefore was designed to provide the first base-line data that compared the quality of some selected antibiotics with their corresponding pharmacopeia. To the best of our knowledge, this type of study has not been conducted in the country of Belize hence its significance to both the Belize Ministry of Health and The University of Belize who currently is the only university in the country responsible for the training of pharmacist.

## Materials

Standardized ruler, Ohaus® Scout™ Pro electronic digital scale (S1 Fig), Monsanto type hardness tester (S2 Fig), Roche^@^Tablet Friability Test Apparatus (single drum) (S3 Fig), and Ajanta^@^ Tablet Disintegration Test Apparatus (double basket) (S4 Fig).

## Methods

### Sampling

A modified Newton et al., (2009) [15] proposed Medicine Quality Assessment Reporting Guidelines (MEDQUARG) was adapted as part of the sampling strategy for this study. The study specifically looked at the question “Are there antibiotics of poor quality in certified drug outlets in Belize?” The study was not designed to conduct complex tests to ascertain quality of the selected oral antibiotics rather an initial screening to determine quality. Based on the MEDQUARG sampling strategy therefore, five brand products of Amoxicillin 500mg, Co-Trimoxazole 960mg and Ciprofloxacin 500mg in oral tablet and/or capsule formulations were conveniently purchased from licensed community pharmacies specifically in San Ignacio Town, Belmopan City and Orange Walk Town and from licensed distributor companies. 90 units of tablets or 60 units of capsules with same batch numbers were selected and packed in sterile specimen containers and coded as follows:

- AMOX C_1_ to C_5_ for each brand of Amoxicillin 500mg capsules,
- CO-TRI T_1_ to T_5_ for each brand of Co-Trimoxazole 960mg tablets,
- CIPRO T_1_ to T_5_ for each brand of Ciprofloxacin 500mg tablets.

Tablets and capsules were purchased locally from verified licensed and registered pharmaceutical stores in the country of Belize. The samples were purchased between the months of July to September 2019.

A sample is considered to fail screening when it failed visual inspection, weight variation, friability test and disintegration test when compared with corresponding pharmacopoeia references.

### Data analysis

Data entry and analysis was done by both Microsoft Excel 2007 [16] and IBM^®^ Statistical Package for Social Sciences (SPSS^®^) Statistics version 25 for Windows [17].

### Storage and transportation

Purchased tablets and capsules were transported in carefully packaged sterile specimen containers with sterile gauze to ensure the protection of samples. All containers were adequately sealed and packaged in such a manner to avoid breakage and/or contamination during transportation and storage [18]. Storage conditions were kept in accordance with the storage requirements for each individual drug. Generally, samples were stored at an ambient temperature of between 69°F (20.56°C) to 77°F (25°C) until tested. The medications were stored at the University of Belize Pharmacy Laboratory under air conditioning, away from sunlight and away from access to other individuals. The samples were only accessible to the researchers when the tests were performed. No other person had access to the samples.

### Visual inspection

Visual inspection refers to the process of identifying crucial container integrity defects such as cracks, misapplied stoppers/seals, unidentified material, precipitation, discoloration and cosmetic defects such as cracks, scratches and dirt [19]. All the packaging and blister packs of formulations were inspected manually and any abnormal spelling or unduly faded colours of the packaging were recorded. The visual inspection process followed International Pharmaceutical Federation (FIP) and the USP tool for Visual Inspection of Medicines. A checklist for visual inspection of medicines was used to identify suspicious products for further examination [20]. Each medication was thoroughly physically examined using the checklist in S1 Appendix. Sizes (length, width and diameter) of dosage form were measured using a standardized ruler to ensure uniformity and results were recorded. Mean was calculated and logged into tables.

### Weight variation

30 tablets of each sample Co-Trimoxazole 960mg and Ciprofloxacin 500mg were weighed, calibrated and measured using an Ohaus® Scout™ Pro electronic digital scale. The results were recorded for each tablet and mean calculated, recorded and compared with corresponding pharmacopeia standards. 30 capsules of each sample Amoxicillin 500mg were weighed whereby whole capsules and empty capsule shells were weighed separately. Powder weights for each sample were also calculated by deducting weight of empty capsule from whole capsule [21, 22]. Results for both capsules and tablets were recorded. The percentage difference in the weight variation was determined within permissible limits.

### Hardness test

The Monsanto type hardness tester was used to test the hardness of each tablet. The tester was first placed across the diameter between the spindle and the anvil. The tablet was then placed in position and the knob adjusted to hold the tablet. Before the pressure was applied, the reading of the pointer was calibrated to zero. The pressure was finally applied slowly to determine the hardness of the tablet by breaking it. The test was measured in kilograms (kg) and later converted to Newton’s (N) as corresponding pharmacopoeia requirement [23, 24]. 30 tablets of each brand Co-Trimoxazole 960mg and Ciprofloxacin 500mg were tested for hardness and average was determined and recorded.

### Friability test

10 sample tablets of each antibiotic were placed in Tablet Friability Test chamber (single drum) to determine broken tablets and the amount lost through chipping. Each brand antibiotic tablets were tested three times at 25 ± 1 Revolutions Per Minute (RPM) at 4 minutes (approximately 100 rotations) [25, 26] and the sample tablets we then weighed and recorded to compare with corresponding pharmacopoeia reference (total of 30 tablets per brand). Friability was calculated using the simple formula:

Friability=[(W1−W2)÷W1]×100

Where W1 = weight of the tablets before test while W2 = weight of the tablet after test

### Disintegration test

Ajanta^@^ Tablet Disintegration Test Apparatus (double basket) was utilized and a water bath was maintained at 37°C ± 2°C whereby apparatus was set to run at 30 minutes with 29 to 32 cycles per minute [27–29]. Results obtained were recorded as mean. Results were then compared with corresponding pharmacopoeia reference(s). Collected brands of Amoxicillin 500mg, Co-Trimoxazole 960mg and Ciprofloxacin 500mg were tested for disintegration using the following procedures:

1unit of each obtained brand antibiotic was placed in each of six tubes (1 round) of each basket in disintegration test apparatus. Every 5 minutes the mesh of each tube was checked for disintegration process and results were logged. Each basket was recorded as one round and a total of 5 rounds were conducted (a total of 30 units for each brand antibiotics). Mean was calculated and comparison made with corresponding pharmacopeia.

## Results and discussion

The arbitrary pharmacopoeial acceptance limits of Amoxicillin 500mg, Co-Trimoxazole 960mg and Ciprofloxacin 500mg were compared in this study with the aim to providing baseline information on the quality of these antimicrobial agents. The general and individual guidelines, and set criteria of the different antibiotics were assessed and compared for quality. These physical quality factors have serious health and economic consequences for the patient and for the country. Moreover, the risk of poor quality antibiotics has detrimental effects on patient’s prognosis, antibiotic resistance and mortality rate [30].

30 units of each sample Amoxicillin 500mg (AMOX C_1_ –C_5_), Co-Trimoxazole 960mg (CO-TRI T_1_ –T_5_) and Ciprofloxacin 50mg (CIPRO T_1_ –T_5_) were tested for weight variation and disintegration for both capsules and tablets formulations. Only hardness and friability tests were conducted for tablet formulation. Table 1 indicates all antibiotic samples collected and tested from different pharmacopoeia standards. Physiochemical parameters of each antibiotics are summarized in Tables 2–4 for Amoxicillin 500mg, Co-Trimoxazole 960mg and Ciprofloxacin 500mg respectively. The detailed results of each antibiotic are discussed under each test performed.

**Table 1:** Pharmacopoeia standards of antibiotics.

Antibiotic	USP	BP
**Amoxicillin 500mg**	AMOX C_1_	AMOX C_3_
	AMOX C_2_	AMOX C_4_
		AMOX C_5_
**Co-Trimoxazole 960mg**	CO-TRI T_4_	CO-TRI T_1_
	CO-TRI T_5_	CO-TRI T_2_
		CO-TRI T_3_
**Ciprofloxacin 500mg**	CIPRO T_1_	CIPRO T_5_
	CIPRO T_2_	
	CIPRO T_3_	
	CIPRO T_4_	

**Table 2:** Physiochemical parameters of different brands of Amoxicillin 500mg capsules.

Code No.	Diameter Mean ± SD (cm)	Thickness Mean ± SD (cm)	Weight Variation Whole capsule Mean ± SD (g)	Weight Variation Powder Mean ± SD (g)	Disintegration time Mean ± SD (min)
AMOX C1	2.10 ± 0.00	0.70 ± 0.00	0.69 ± 0.0281	0.59 ± 0.0287	30 ± 0.00
AMOX C2	2.30 ± 0.00	0.70 ± 0.01	0.72 ± 0.0083	0.62 ± 0.0083	30 ± 0.00
AMOX C3	2.20 ± 0.00	0.80 ± 0.00	0.67 ± 0.0096	0.57 ± 0.0103	19 ± 5.48
AMOX C4	2.20 ± 0.00	0.80 ± 0.00	0.67 ± 0.0194	0.58 ± 0.0208	20 ± 7.91
AMOX C5	2.40 ± 0.00	0.70 ± 0.00	0.70 ± 0.0130	0.59 ± 0.0123	20 ± 7.07

**Table 3:** Physiochemical parameters of different brands of Co-Trimoxazole 960mg tablets.

Code No.	Diameter Mean ± SD (cm)	Width Mean ± SD (cm)	Thickness Mean ± SD (cm)	Weight Variation Mean ± SD (g)	Hardness Mean ± SD (N)	Friability Mean ± SD (%)	Disintegration Time Mean ± SD (min)
CO-TRI T1	1.88 ± 0.04	0.90 ± 0.00	0.50 ± 0.00	1.02 ± 0.0131	109.34 ± 21.6487	0.91 ± 0.3984	2.00 ± 0.00
CO-TRI T2	1.90 ± 0.00	0.90 ± 0.00	0.60 ± 0.00	1.20 ± 0.0122	118.01 ± 22.1141	0.19 ± 0.0462	3.50 ±0.50
CO-TRI T3	1.90 ± 0.00	0.90 ± 0.00	0.70 ± 0.02	1.03 ± 0.0045	122.09 ± 12.6838	0.35 ± 0.0577	1.90 ± 0.14
CO-TRI T4	1.91 ± 0.02	0.90 ± 0.00	0.70 ± 0.00	1.19 ± 0.0099	125.85 ± 10.6444	0.14 ± 0.0520	2.50 ± 0.50
CO-TRI T5	1.90 ± 0.00	0.80 ± 0.01	0.60 ± 0.01	1.02 ± 0.0192	110.00 ± 28.0401	0.46 ± 0.2042	25 ± 11.18

**Table 4:** Physiochemical parameters of different brands of Ciprofloxacin 500mg tablets.

Code No.	Diameter Mean ± SD (cm)	Width Mean ± SD (cm)	Thickness Mean ± SD (cm)	Weight Variation Mean ± SD (g)	Hardness Mean ± SD (N)	Friability Mean ± SD (%)	Disintegration time Mean ± SD (min)
CIPRO T1	2.00 ± 0.00	0.90 ± 0.00	0.45 ± 0.00	0.97 ± 0.0138	91.77 ± 19.3867	0.65 ± 0.2138	4.40 ± 0.89
CIPRO T2	1.60 ± 0.00	0.80 ± 0.00	0.51 ± 0.02	0.68 ± 0.0212	109.02 ± 22.0765	0.05 ± 0.0866	5.80 ± 0.45
CIPRO T3	1.90 ± 0.00	0.80 ± 0.00	0.50 ± 0.00	0.87 ± 0.0121	123.24 ± 13.6569	0.46 ± 0.1150	10.80 ± 10.73
CIPRO T4	1.90 ± 0.00	0.90 ± 0.00	0.50 ± 0.00	0.93 ± 0.0088	109.43 ± 26.8526	0.14 ± 0.0577	2.95 ± 0.11
CIPRO T5	1.70 ± 0.00	0.80 ± 0.00	0.50 ± 0.00	0.69 ± 0.0079	132.23 ± 12.8111	0.05 ±0.0808	8.00 ±0.00

### Visual inspection

Great importance is given to visual inspection of dosage forms, since it frequently provides a first vital indication of degradation, poor manufacturing, tampering or counterfeiting [31]. The powdered surfaces, the non-uniform scoring depths, and the indentations observed on the tablets in this study are indications that further testing is required to identify the problem, which could either be from manufacturing practices or from transportation and storage. Degradation during storage and transportation is of particular significance especially in tropical countries like Belize [32].

### Amoxicillin 500mg

The checklist (S1 Table) for visual inspection was used to inspect all the sample brands of AMOX C_1_ –C_5_ collected. Apart from AMOX C_2_ as loose capsules, AMOX C_1_, C_3_, C_4_ and C_5_ were packaged individually in blister packs. AMOX C_1_ and C_5_ were packaged in aluminum with transparent polyvinyl chloride (PVC) whereas AMOX C_3_ and C_4_ in aluminum with non-transparent (white) PVC. In terms of labeling, and trade names where applicable, spelling and information provided (either in English or Spanish) were appropriate. AMOX C_1_–C_5_ were observed to have uniform size with standard deviation (SD) of 0 in cm for diameter and thickness as shown in Table 2. Printing behind blister packs showed sign of fading for AMOX C_1_ especially when rubbed, but words were still evident. Raw Shape, color and texture, were also uniform and samples were free of contamination. However, a few empty capsules had minimal powder remnants on PVC after capsules were removed from original package (AMOX C_1_ and C_4_), indicating spillage. The results from the present study suggests more in-depth tests to be conducted to ensure quality of AMOX C_1_ and C_4_.

#### Co-Trimoxazole 960mg

The checklist (S2 Table) for visual inspection was also used to inspect all the sample brands of CO-TRI T_1_ to T_5_ collected. CO-TRI T_2_ and T_4_ were packaged in loose tablets while CO-TRI T_1_, T_3_ and T_5_ were packaged individually in blisters. CO-TRI T_1_ and T_3_ were packaged in aluminum with transparent PVC while CO-TRI T_5_ was packaged in aluminum with semi-transparent (yellow) PVC. Information provided on labeling, trade names, and spelling was appropriate for the tested samples. The results shown in Table 3 indicated that all CO-TRI T_1_ to T_5_ have uniform size with SD between 0.00 and 0.04 in cm for diameter, width and thickness. Shape, color, texture, and tablet markings were all uniform and samples were free of contamination. Minimal powders on PVC were also noted to be present in all samples. Thus, more complex tests may be required to ensure quality of CO-TRI T_1_ –C_5_ as this may be due to the kind of coating used which may indicate a possible fault in manufacturing, storage or transportation [31]. Additionally, few chippings were also observed in CO-TRI T_1_ and T_5_. Though chippings were observed, friability test results were found to be within acceptable limits.

#### Ciprofloxacin 500mg

Furthermore, the checklist (S3 Table) for visual inspection was used to inspect all samples of Ciprofloxacin 500mg tablets. CIPRO T_1_ was packaged in aluminum backing and covers (ALU-ALU), while CIPRO T_2,_ T_3,_ and T_5_ were packaged in aluminum with transparent PVC. Likewise, CIPRO T_4_ was packaged in aluminum with semi-transparent (brown) PVC. Appropriate labeling, trade names, and spelling information was provided for CIPRO T_1_ to T_5_ samples. The results shown in Table 4 indicated that all CIPRO T_1_ to T_5_ have uniform size with SD 0.00 and 0.02 in cm for diameter, width and thickness. Shape, color, texture, and tablet markings were also uniform, and samples were free of contamination in CIPRO T_4_ and T_5_. Slight chipping was evident in CIPRO T_1_ and tablet surface was noted to be powdered and scoring depths were not uniform. Tablet surface from CIPRO T_2_ appeared to be uneven and pinholes were noted to be present. Color was not uniform in CIPRO T_3_ and minimal powder was present on blister after tablets were removed from original package. Therefore, more detailed examination and tests will be needed for CIPRO T1, T2 and T3 as powdered surfaces, the non-uniform scoring depths and the indentations on the tablets are indicators that further testing is required to identify the problem either from manufacturing practices or from transportation and storage. This is important to note as degradation during storage and transportation is of particular significance especially in tropical countries like Belize [32].

### Weight variation test

Weight uniformity is very important as it ensures that consumers take a precise pharmaceutical dose. Furthermore, weight uniformity ensures that a consistent dose and quantity of API is maintained between all batches and doses [33]. The fluctuations in the weight variation seen in some of the samples may indicate poor quality control measures either by inconsistent powder, granulate density or particle size distribution, which are all common sources of weight variation during compression. Regardless of the reason, the tablets, though under the acceptable range may provide sub-therapeutic levels of the antibiotics which in turn may contribute to antibiotic resistance. Similarly, the tablet samples found to weigh over the acceptable range can negatively impact the patient through increased adverse effects, increased toxicity levels and increased potential for drug-drug interactions.

Under pharmacopeia standards, tablets/capsules over 249mg weight for BP standards and 324mg for USP standards should not deviate 10% from average weight, and no more than 2 tablets/capsules should deviate from average weight from a test of 20 sample tablet/capsules [19, 21, 34].

#### Amoxicillin 500mg

30 capsules of each brand were weighed by whole capsule, powder and empty capsule, and logged respectively in grams (S4 Table and S5 Table). They capsules were then examined against their corresponding pharmacopeia standards, either USP or BP.

**Fig 1:**
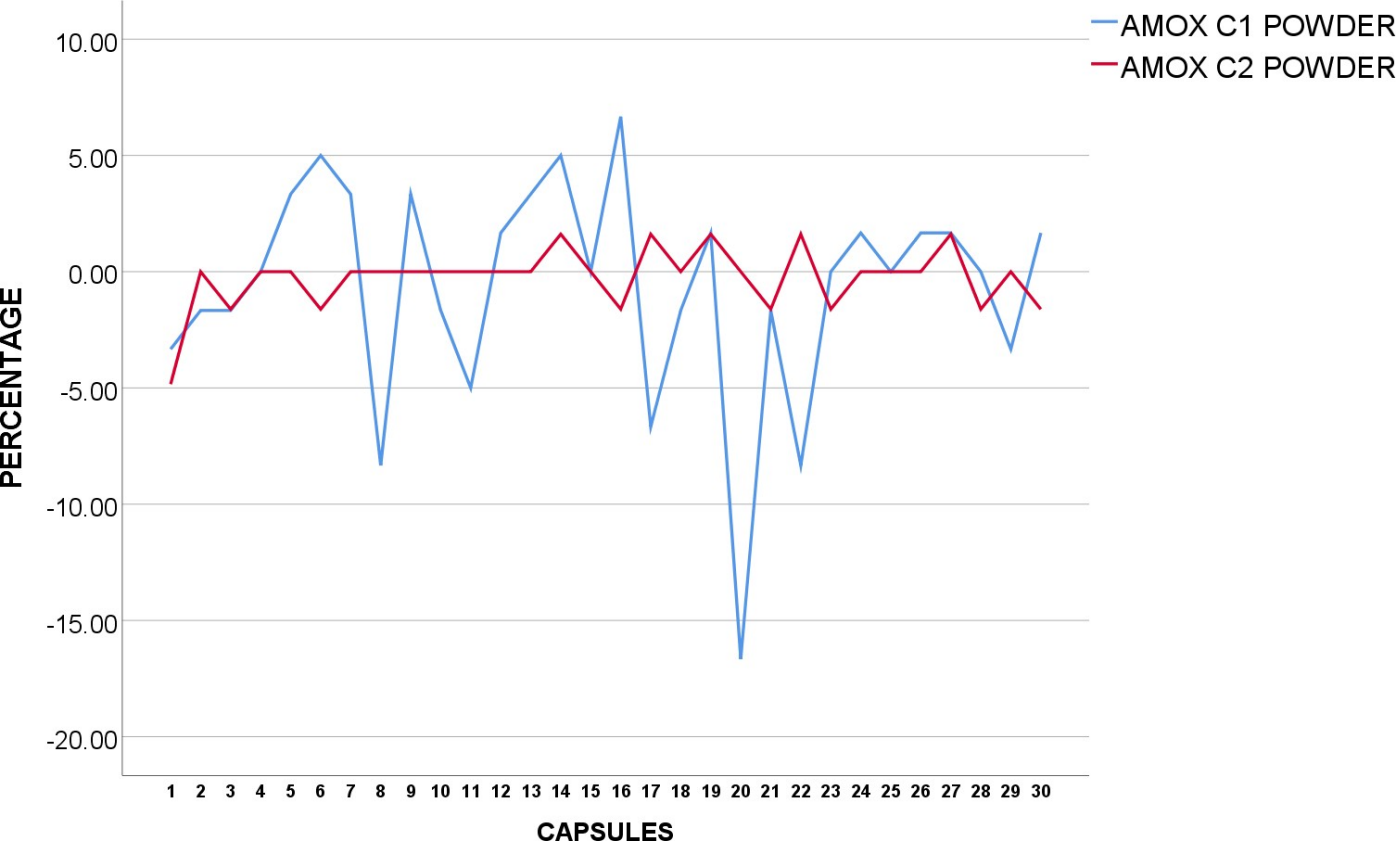
Weight uniformity of USP Amoxicillin 500mg from mean (%).

AMOX C_1_ and C_2_ tested samples were under USP standards. AMOX C_1_ failed to be within standard acceptable range. Weight variation for both whole capsules and powder of AMOX C_1_ for three capsules were noted to be below 5% average weight, and one capsule was over 5% average weight. Another capsule was found to be below 10% average weight as shown in Fig 1 below. The higher SD value of 0.0281 powder weight in grams in AMOX C_1_ (Table 2) further reflects the results. AMOX C_2_ has been noted to be within acceptance value under USP standards as shown in Fig 1. None of capsules were found to be over or under 5% average weight.

**Fig 2:**
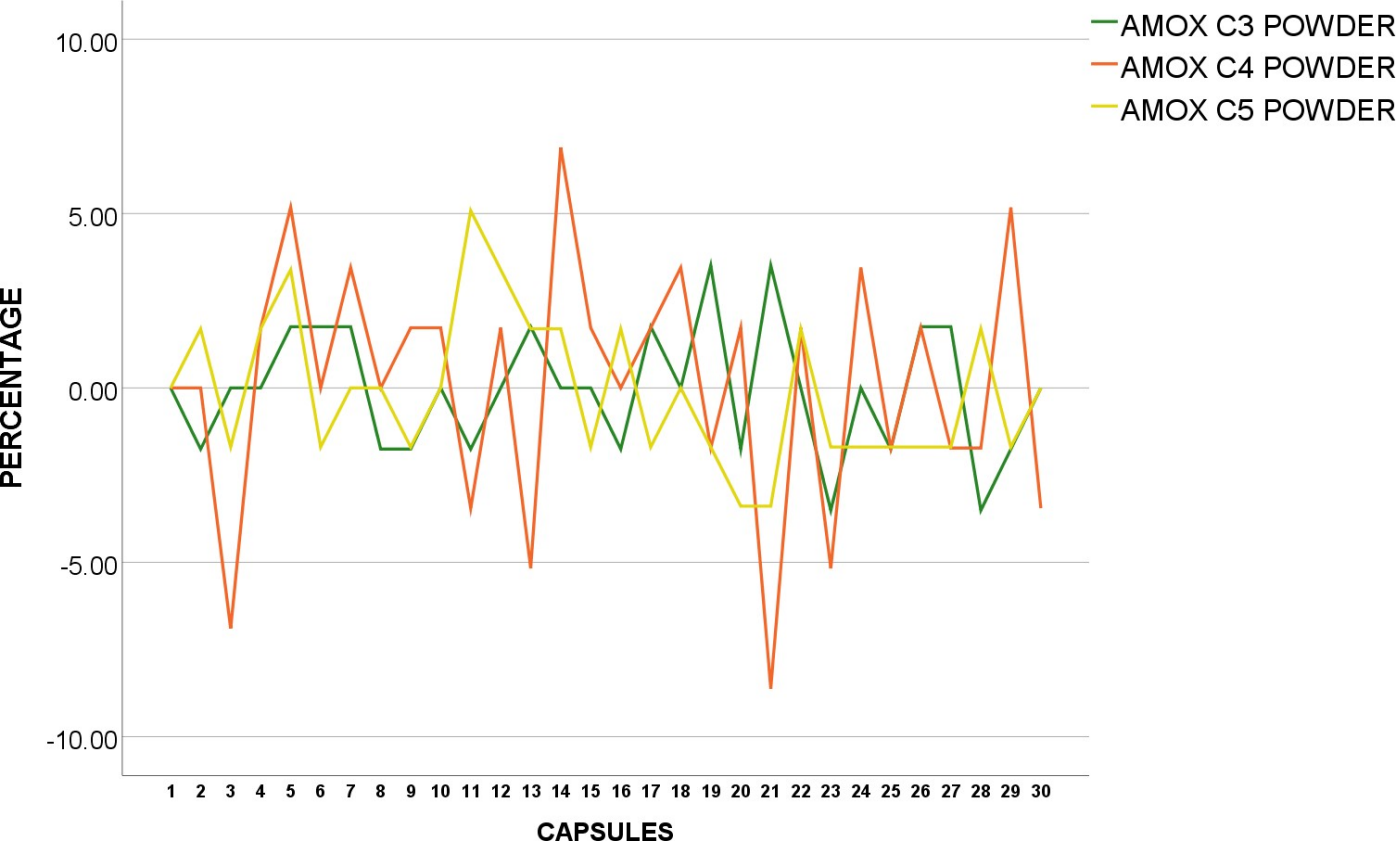
Weight uniformity of BP Amoxicillin 500mg from mean (%).

AMOX C_3_ conformed to BP standards as none of the 30 capsules were over or under 5% from average weight as shown in Fig 2. For AMOX C_4_, the weights of four powder sample were found to be under and three samples were over 5% of average weight as shown in Fig 2. Nevertheless, none of the weights had over or below 10% from average weight in AMOX C_4_ with a higher SD at 0.0194 powder weight in grams. Only one of the powder sample was found to be slightly over 5% of average weight in AMOX C_5_. The results of the tests for AMOX therefore, showed that AMOX C_3_ and C_5_ conformed to BP weight variation standards, while AMOX C_4_ failed to comply with weight uniformity according to corresponding standards. More than 2 individual capsules and powder samples were shown to be over 5% of average weight. Detailed examination and testing is recommended for AMOX C_1_ and C_4_ as both failed to pass corresponding pharmacopeia references in terms of weight variation.

#### Co-Trimoxazole 960mg

30 tablets of each brand of CO-TRI were weighed and logged in grams (S6 Table). All five brands of Co-Trimoxazole 960mg tested have a SD of 0.0045 and 0.0192 in grams as shown in Table 3.

**Fig 3:**
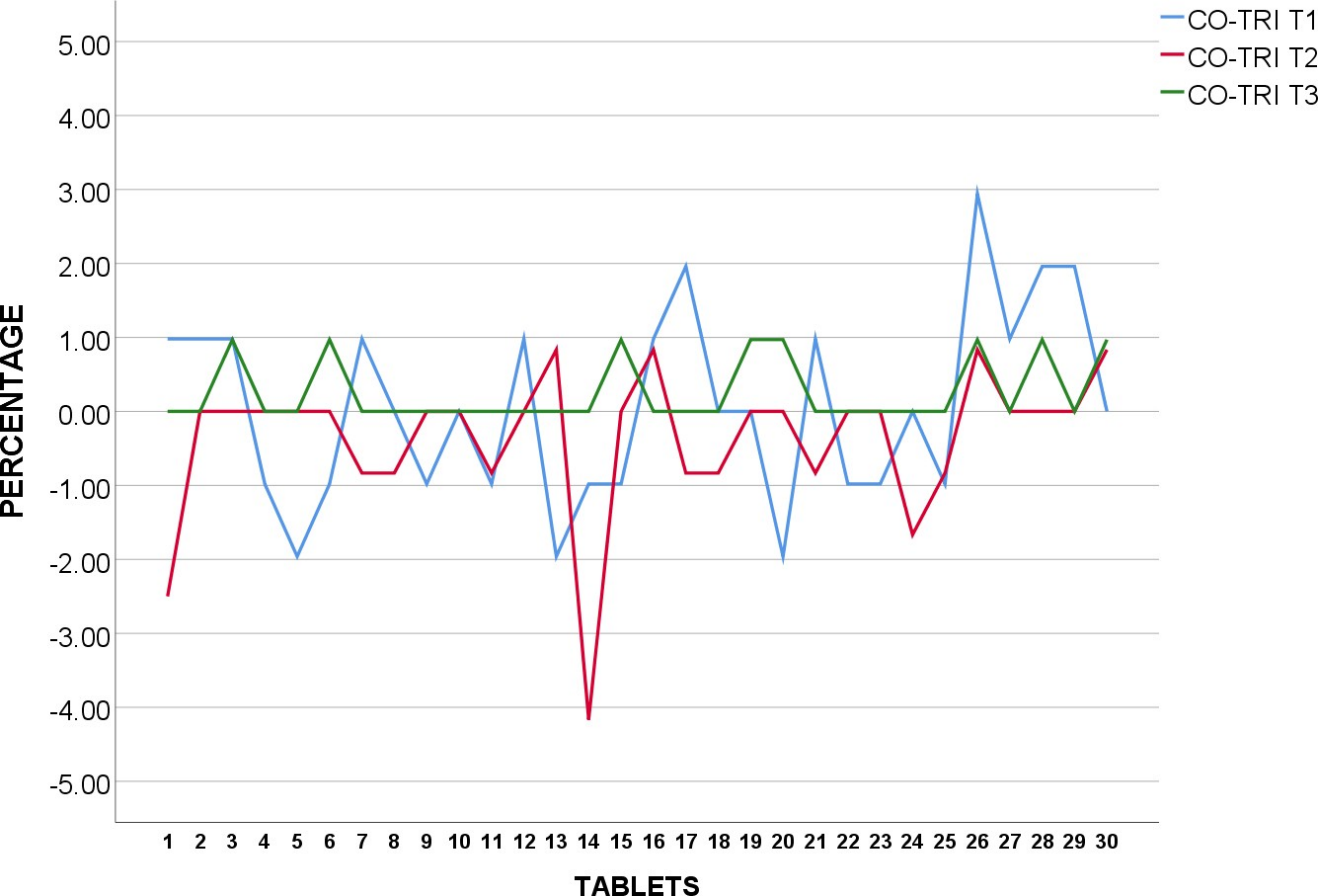
Weight uniformity of BP Co-Trimoxazole 960mg from mean (%).

The weight of 30 tablets each of CO-TRI T_1_, T_2,_ and T_3_ were analysed and found to conform to BP standards as none of the 30 tablets were over or under 5% from average weight as shown in Fig 3.

**Fig 4:**
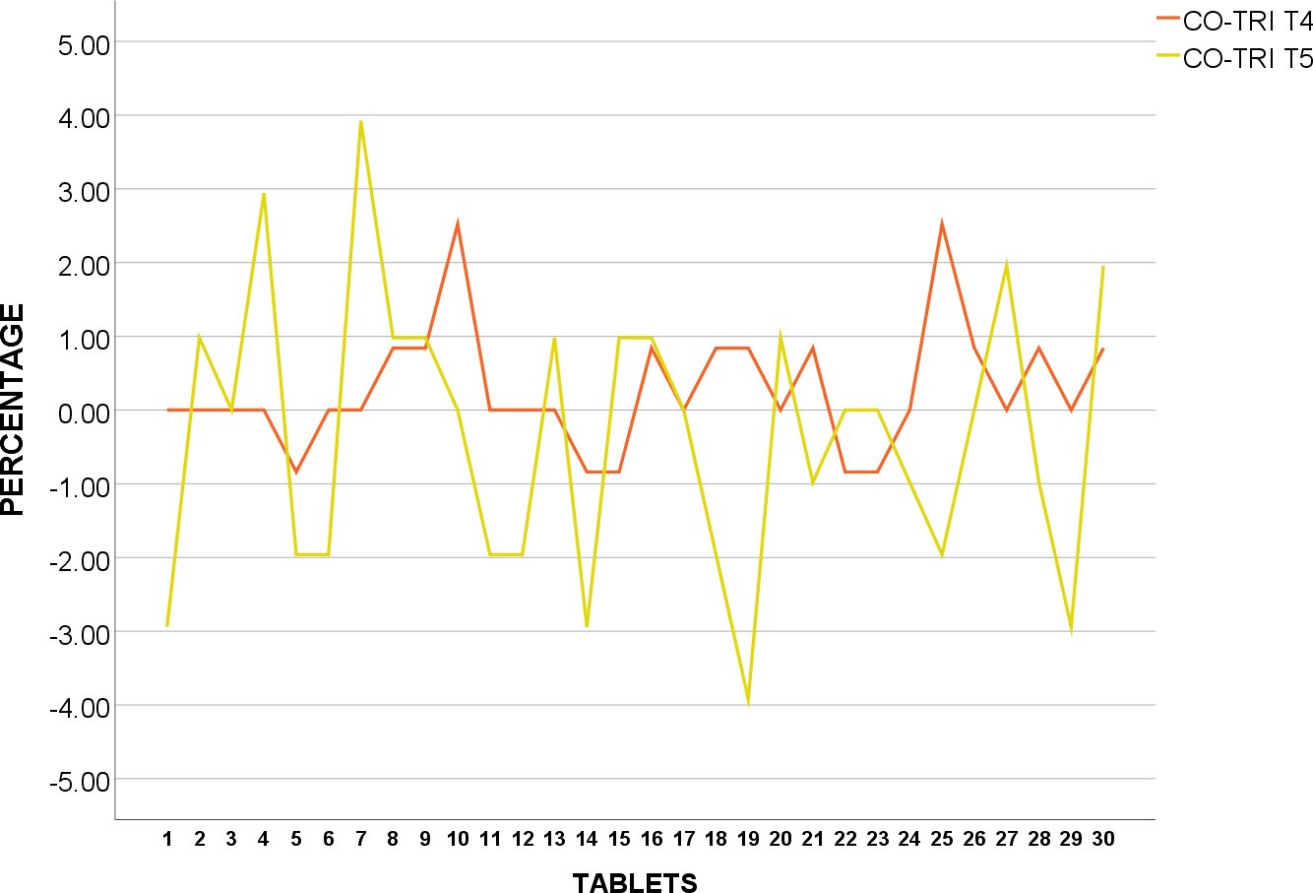
Weight uniformity of USP Co-Trimoxazole 960mg from mean (%).

CO-TRI T_4_ and T_5_ were also tested for weight uniformity, and found to be within acceptance value under USP pharmacopoeia standards as none of the tablets were over or under 5% from average weight as shown in Fig 4.

All samples passed corresponding pharmacopeia references in terms of weight variation. This may be an indication of constant levels of API, thereby preventing fluctuation of systemic API [33].

#### Ciprofloxacin 500mg

30 tablets of each brand were weighed and logged in grams (S7 Table). All five brands of Ciprofloxacin 500mg tested had weight variation test showing a SD 0.0079 and 0.0212 in grams. CIPRO T2 was noted to have higher SD than others at 0.0212 in grams (Table 4). These tablets were later used in Tablet hardness test.

**Fig 5:**
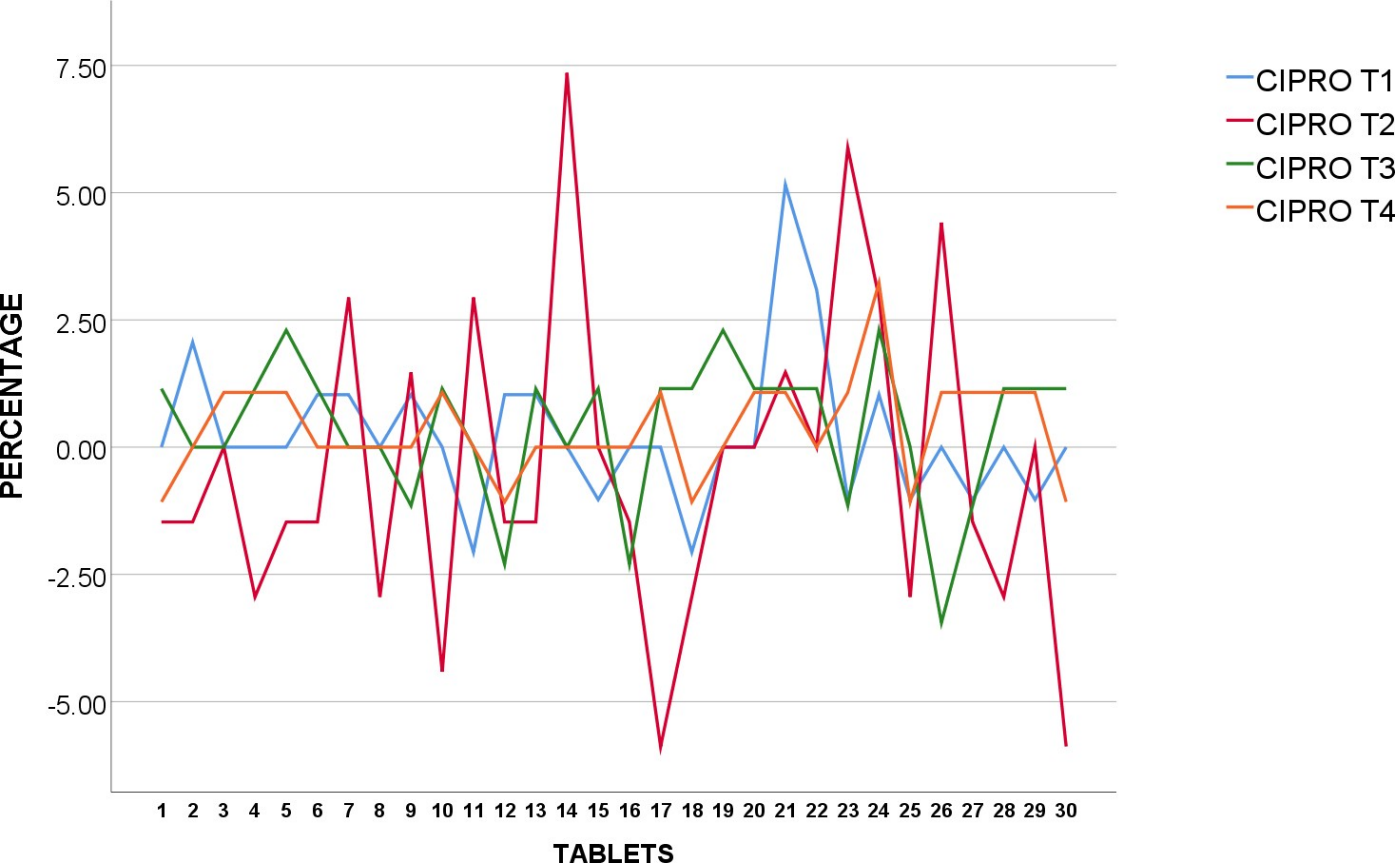
Weight uniformity of USP Ciprofloxacin 500mg from mean (%).

CIPRO T_1_, T_3,_ and T_4_ were found to be within acceptable value under USP standards as none of the tablets were over or under 5% from average weight as shown in Fig 5. However, two tablets were found to be over, and two tablets under 5% average weight in CIPRO T_2_, which reflected upon the higher SD as earlier observed, none deviated 10% over or under average weight.

**Fig 6:**
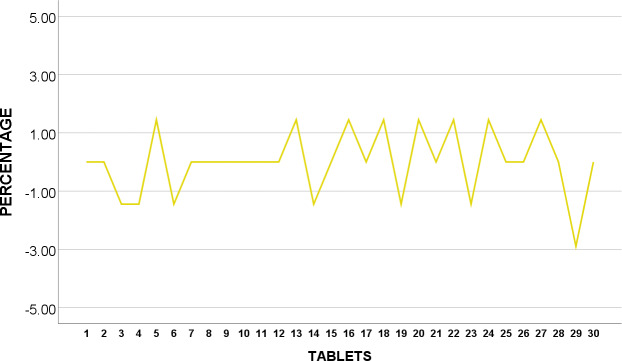
Weight uniformity of BP Ciprofloxacin 500mg from mean (%).

The weight of 30 tablets CIPRO T_5_ were analysed and shown to be in conformity with BP standards as none of the 30 tablets were over or under 5% from average weight as revealed in Fig 6.

We suggest further testing and examination of CIPRO T_2_ as they were found to fail corresponding specification in weight variation test. This may indicate variation of API or even presence of impurities whereby patients may run the risk of having drug-drug interactions, toxicity and even treatment failures [33].

### Hardness test

Tablet hardness serves both as a criterion to guide product development and as a quality-control specification. To this end, tablets that are too hard could be due to excessive bonding potentials between active ingredients and excipients thereby preventing proper dissolution. On the contrast, tablets that are too soft could be due to weak bonding which subsequently leads to premature disintegration or chipping and breaking [35]. Both cases are counterproductive, and as observed in the present results, most samples have high standards of deviation of the hardness test. This may lead to some of the tablets being broken down and dissolved before it reaches its absorption site, meanwhile the latter may pass undissolved through its absorption site hence inhibiting the tablet to perform its pharmacological activity. The inconsistent readings may lead to drastic variations in bioavailability in between doses. Monsanto type hardness tester was utilized for testing and results were initially in kilogram (kg) which were later converted to N as per BP and USP standards (1 kg = 9.8066500286389 N) [23, 24].

#### Co-Trimoxazole 960mg

**Fig 7:**
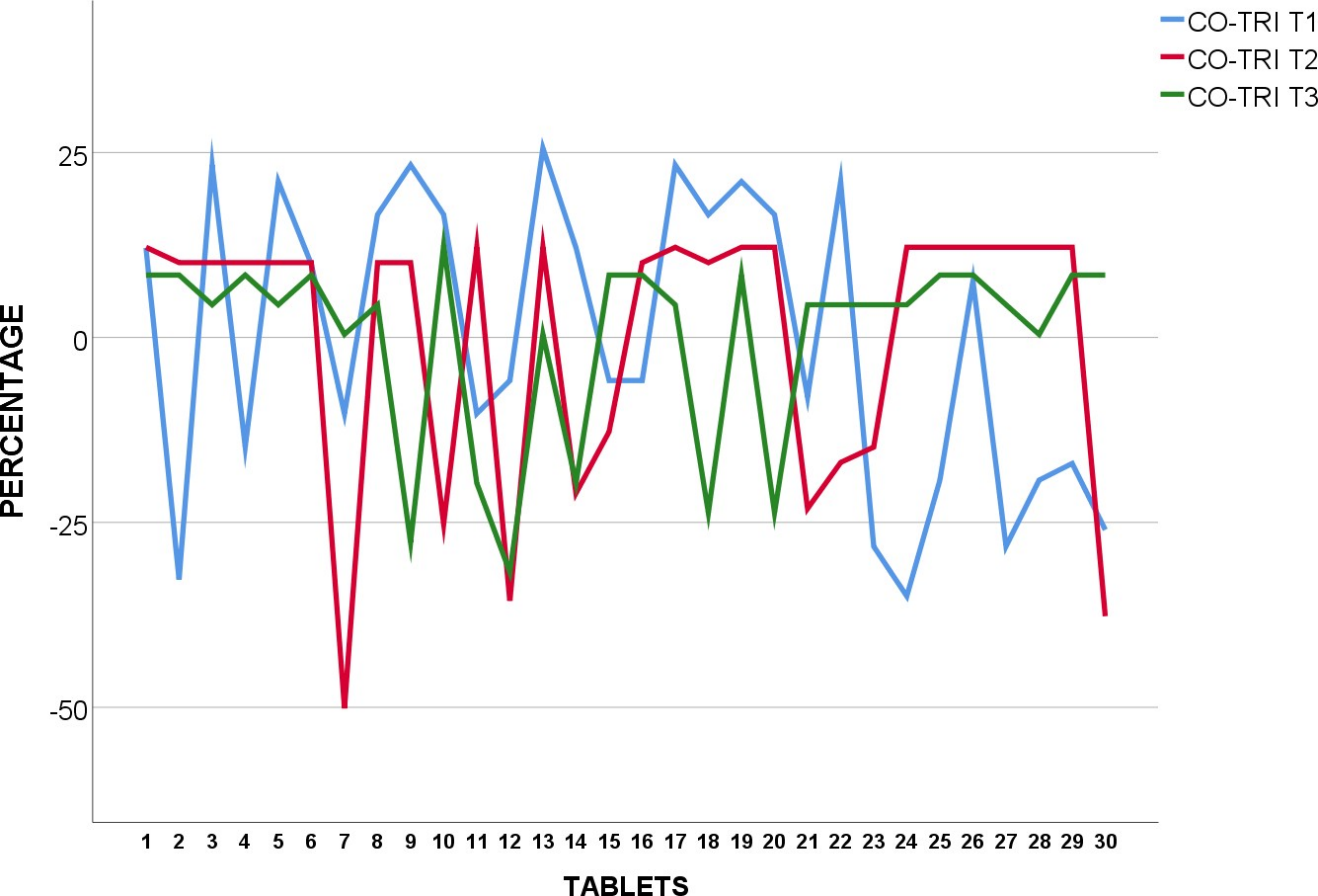
Hardness variation of BP Co-Trimoxazole 960mg from mean (%).

**Fig 8:**
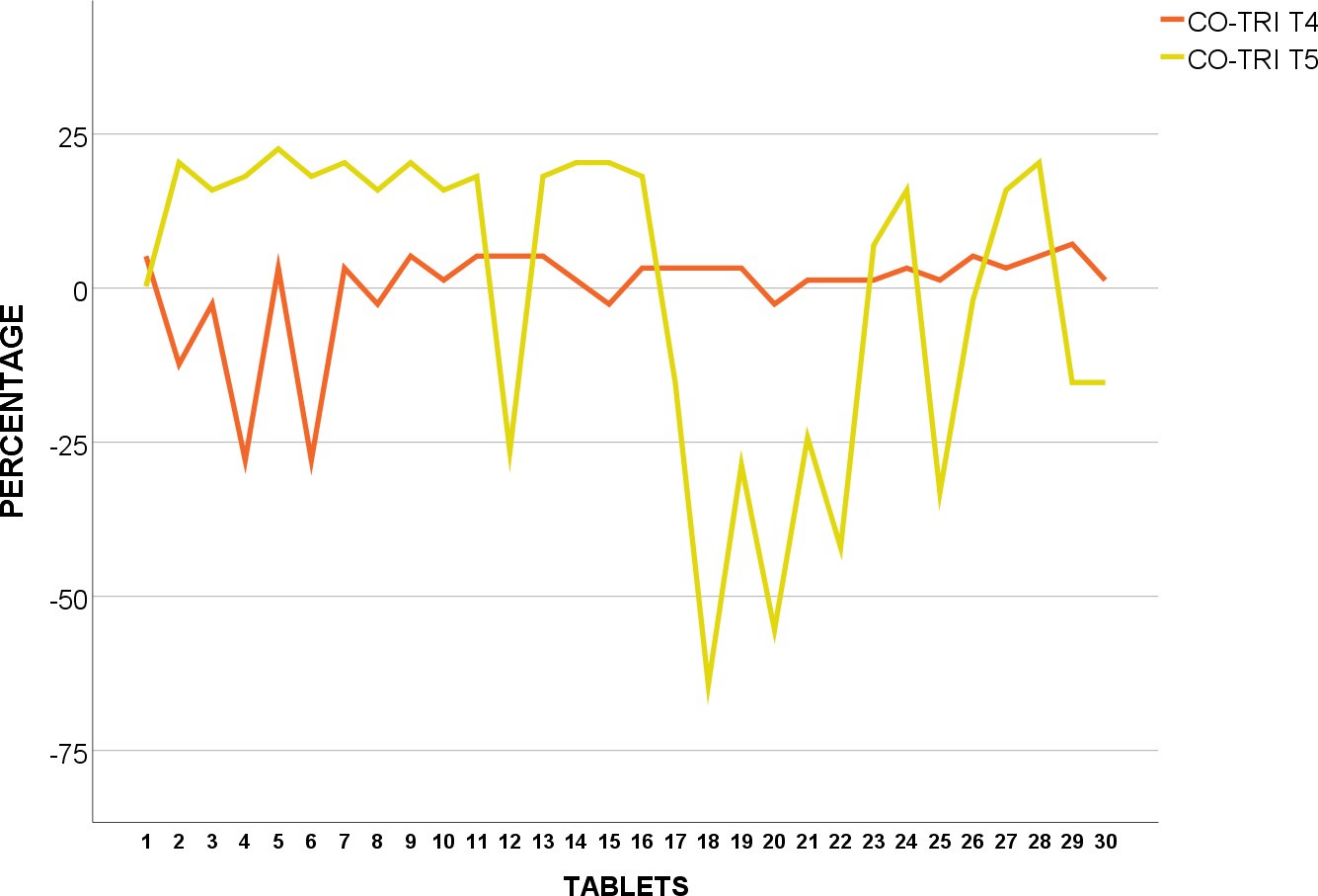
Hardness variation of USP Co-Trimoxazole 960mg from mean (%).

The breaking force of a tablet is a form of measuring mechanical integrity [24]. Hardness test results appeared to be inconsistent, especially for CO-TRI T_1_, T_2_ and T_5_ with high SD of 21.6487, 22.1141 and 28.0401 in N respectively (Table 3). Figs 7 and 8 presents the percentage hardness variation from average under BP and USP respectively. CO-TRI T_1_ –T_5_ were shown to be inconsistent in tablet hardness which may indicate drastic bioavailability in vivo between doses [35]. However, as hardness test is one of which determines whether tablets may disintegrate, the batch is still accepted if disintegration test are within specified range [35]. Therefore, these batches cannot be deemed pass or fail without performing disintegration test.

#### Ciprofloxacin 500mg

**Fig 9:**
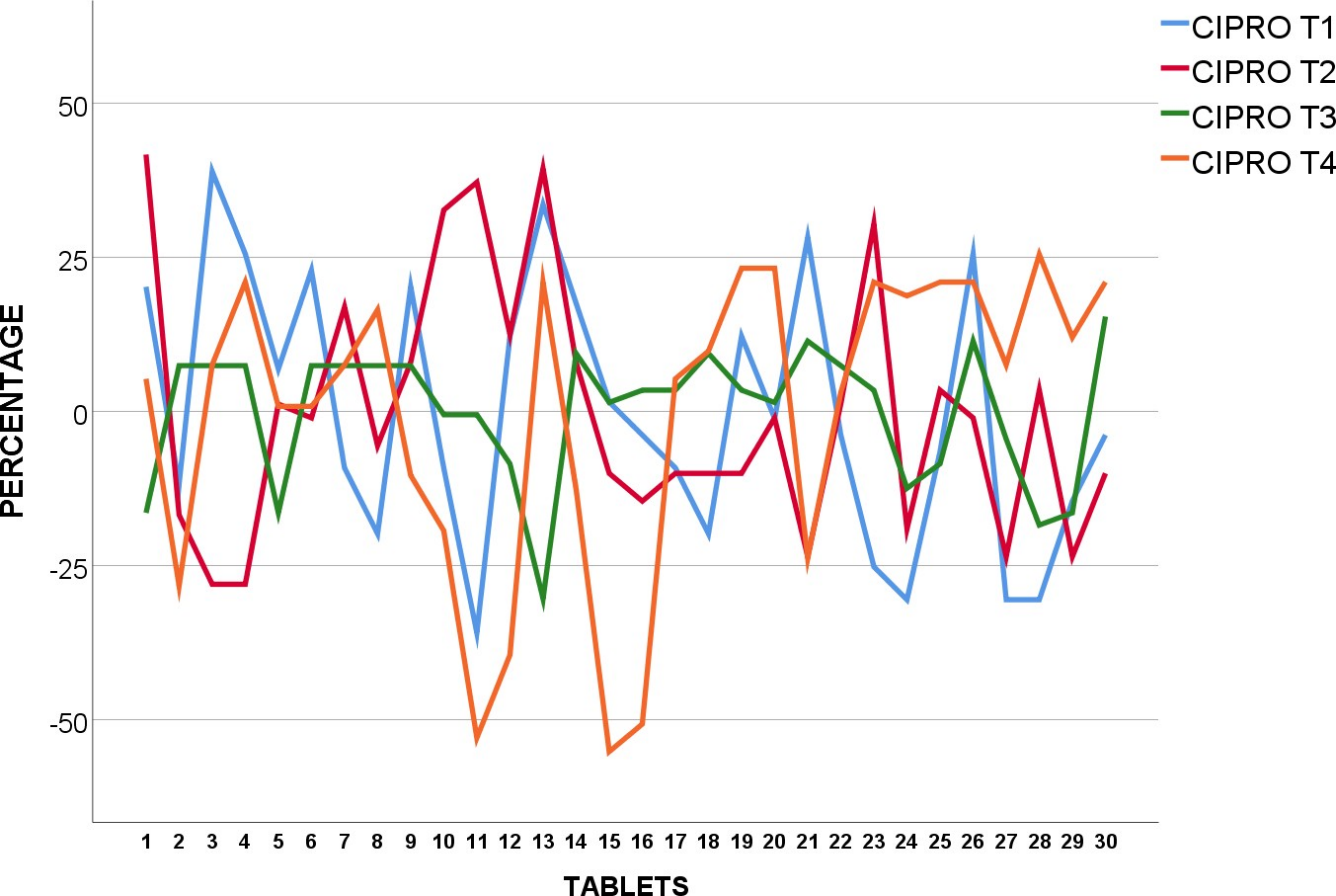
Hardness variation of USP Ciprofloxacin 500mg from mean (%).

**Fig 10:**
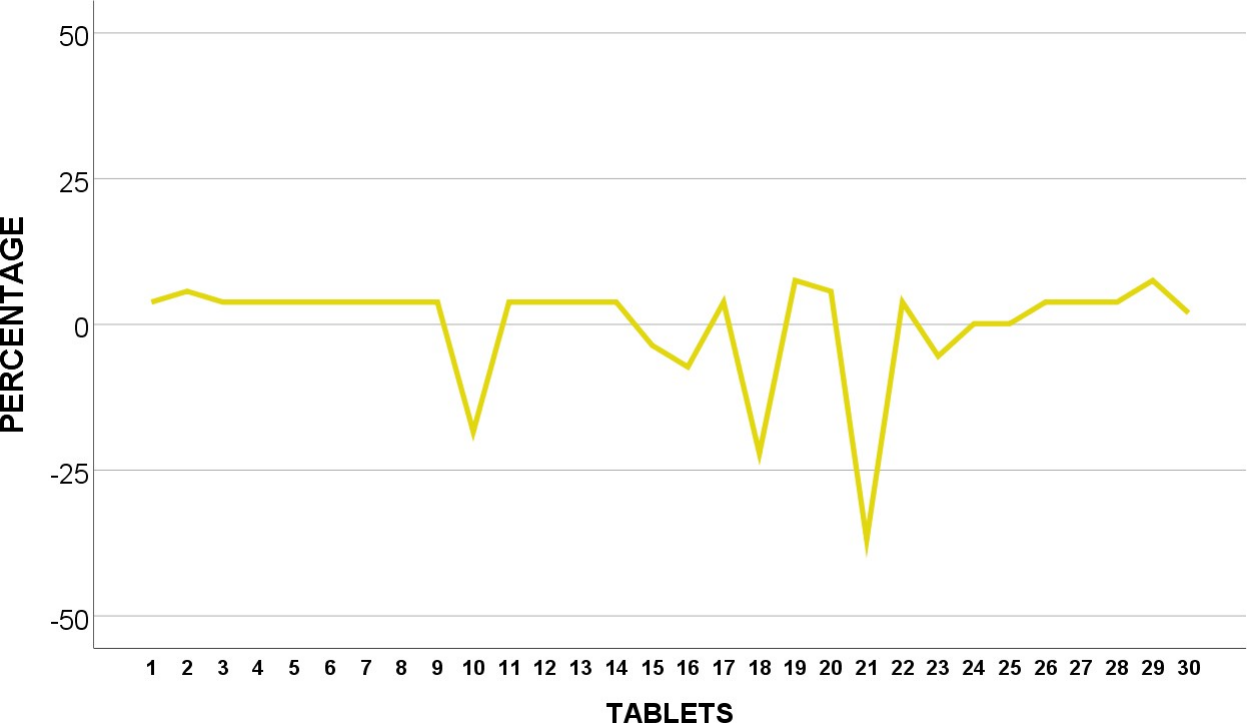
Hardness variation of BP Ciprofloxacin 500mg from mean (%).

Hardness test results appeared to be inconsistent, especially in CIPRO T_2_ and T_4_ with high in N of 22.0765 and 26.8526 respectively (Table 4) which may indicate drastic bioavailability in vivo between doses as shown in Fig 9 for USP and Fig 10 for BP [35]. Even so, hardness test cannot be a sole determinant of the acceptability of batch without disintegration test [35]. Therefore, these batches cannot be deemed pass or fail without performing disintegration test.

Even though all tablet samples tested for tablet hardness were shown to be inconsistent, they were within acceptable standards in terms of friability and disintegration tests. Therefore, the batches tested are considered to be acceptable.

### Friability test

Friability testing is another test that determines physical strengths of tablet formulations [25]. Under BP and USP standards, the maximum acceptance loss of mass is 1% (single test or mean of tests) from 10 tablets at 25 ± 1 rotations per minute at 4 minutes (approximately 100 rotations) which can be repeated up to 3 times [25, 26].

#### Co-Trimoxazole 960mg

Total of 30 tablets of each brand Co-Trimoxazole 960mg were tested for friability at 10 tablets per test, and mean of each brand was calculated. Results for each time and percentage loss are shown in S8 Table for those that complied with BP and S9 Table for USP. Mean percentage losses of CO-TRI T_1_ –T_5_ were documented in Table 3.

CO-TRI T_1_ –T_5_ were shown to have less than 1% mean loss as shown in Table 3. This indicates that these antibiotics are able to withstand normal transportation and handling conditions without considerable breaking and/or chipping as to affect formulation [35].

#### Ciprofloxacin 500mg

Similarly, a total of 30 tablets of each brand Ciprofloxacin 500mg were tested for friability at 10 tablets per test and mean of each brand was calculated. Each result for time and percentage loss are shown in S10 Table for those that complied with USP and S11 Table for BP. Mean percentage losses of CIPRO T_1_ –T_5_ were documented in Table 4.

CIPRO T_1_ –T_5_ were shown to have less than 1% loss as shown in Table 4. The results showed that all tablets tested for Ciprofloxacin 500mg are formulated within standards as they were observed to withstand regular transportation and handling conditions. This signifies that tablets of the same batches will not chip or break before they reach consumers and affect efficacy [35].

### Disintegration test

Disintegration test monitors how a dosage form is dispersed [27, 29]. According to USP and BP standards, hard gelatin capsules, regular coated or film coated tablets should disintegrate completely in a water bath maintained at 37 ± 2°C within 30 minutes at 29 to 32 cycles per minute [27–29]; only fragment of capsule shells may remain in the mesh [29].

#### Amoxicillin 500mg

Disintegration time for AMOX C_1_– C_5_ were observed to be within 30 minutes as shown in Table 2. Also, fragments of empty shells were found to be present in AMOX C_1_ and C_2_ at 30 minutes. This indicates that all brands passed disintegration test in accordance to their corresponding pharmacopeia reference.

#### Co-Trimoxazole 960mg

Time required to dissolve CO-TRI T_1_ –T_5_ is shown in Table 3. Apart from CO-TRI T_5_ with higher SD of 11.18 in minutes from mean time, CO-TRI T_1_ to T_4_ had SD of 0.00 to 0.50 in minutes. From the mean disintegration times for CO-TRI T_1_ –T_5_ shown in Table 3, all samples had disintegration time within 30 minutes. Since CO-TRI T_1_ –T_5_ conformed to their corresponding pharmacopeia standards, the results therefore suggests that the tablets are appropriately formulated to disintegrate in vivo and the release of active ingredients will not be delayed, as would be the case if any were not to disintegrate within specified time [36].

#### Ciprofloxacin 500mg

Disintegration time for CIPRO T_1_ –T_5_ is shown in S12 Table. Mean disintegration test of CIPRO T_1_– T_5_ were calculated and reported. Apart from CIPRO T_3_ with higher SD of 10.73 in minutes from mean time, CIPRO T_1_, T_2_, T_4_ and T_5_ had SD of 0.00 to 0.89 in minutes (Table 4). Table 4 demonstrates mean disintegration time for CIPRO T_1_ –T_5_ and, all samples had disintegration time within 30 minutes. Disintegrated within pharmacopeia standards was seen in all tablets tested for disintegration of Ciprofloxacin 500mg, indicating all tablets of the same batch can disperse in vivo within desired time to give pharmaceutical effect [36].

## Conclusion

In developing countries such as Belize, poor quality of drugs is attributed to insufficient quality assurance, poor or substandard storage facilities, a deficiency in or lack of active regulatory systems in place to effectively evaluate drug quality. Presently, in Belize, the drug regulatory system and quality assurance is just developing. Also, as far as we know, little or no research has been conducted in this area hence the need for this baseline study on drug quality. As a baseline study, the physical qualities of selected oral antibiotics in Belize were tested and compared with their corresponding pharmacopeia. Majority of the selected antibiotics passed performed tests when compared with their pharmacopeia. Only a few samples from both BP and USP antibiotics failed the test conducted. The results of the present study provide the need for detailed, regular and consistent quality assurance test for all medications imported to Belize for public consumption. Any test that failed quality, even if only in one parameter, is a clear caution that potential unsuitability of the drug may exist. Since the tests performed in this study were only level 2 of field-based screening of quality assurance [37], we cannot draw a generalized conclusion based on our findings. We therefore recommend a full analytical quality assurance tests using instruments such as High Performance Liquid Chromatography (HPLC) be carried out for quality control that meet international standards.

### Limitations

The main limitation to this study is the number of tests of conducted on the samples collected which in our opinion was not adequate for a wider generalization. Also, we acknowledge that more quality control test for both tablets and capsules could have been done to support current findings. Additionally, the lack of adequate equipment and funding for a more complex drug quality testing were also considered legitimate limitations to the current study. However, since the objective of the study was to conduct a level 2 field-based screening of the antibiotics with the intent to provide a baseline data for use in planning a much larger study, we believe this objective have been adequately achieved, especially that to the best of our knowledge and after careful search on the Internet a similar study have not been conducted in Belize. This therefore makes the study unique and relevant, hence its strength.

## Supporting information

S1 Fig Ohaus® Scout™ pro electronic digital scale. (TIF)

S2 Fig Monsanto type hardness tester. (TIF)

S3 Fig Roche^@^Tablet friability test apparatus (single drum). (TIF)

S4 Fig Ajanta^@^ Tablet disintegration test apparatus (double basket). (TIF)

S1 Appendix Visual inspection checklist. (PDF)

S1 Table Visual inspection summary of different brands of Amoxicillin 500mg capsules. (DOCX)

S2 Table Visual inspection summary of different brands of Co-Trimoxazole 960mg tablets. (DOCX)

S3 Table Visual inspection summary of different brands of Ciprofloxacin 500mg tablets. (DOCX)

S4 Table Weight uniformity of USP Amoxicillin 500mg capsules. (DOCX)

S5 Table Weight uniformity of BP Amoxicillin 500mg capsules. (DOCX)

S6 Table Weight uniformity of Co-Trimoxazole 960mg. (DOCX)

S7 Table Weight uniformity of Ciprofloxacin 500mg. (DOCX)

S8 Table Friability test for BP Co-Trimoxazole 960mg tablets. (DOCX)

S9 Table Friability test for USP Co-Trimoxazole 960mg tablets. (DOCX)

S10 Table Friability test for USP Ciprofloxacin 500mg tablets. (DOCX)

S11 Table Friability test for BP Ciprofloxacin 500mg tablets. (DOCX)
