# Supplementary figures and images for: Field-based screening of selected oral antibiotics in Belize
Source: PLoS One. 2020 Jun 17;15(6):e0234814. doi: 10.1371/journal.pone.0234814 (PMC7299385; doi:10.1371/journal.pone.0234814)

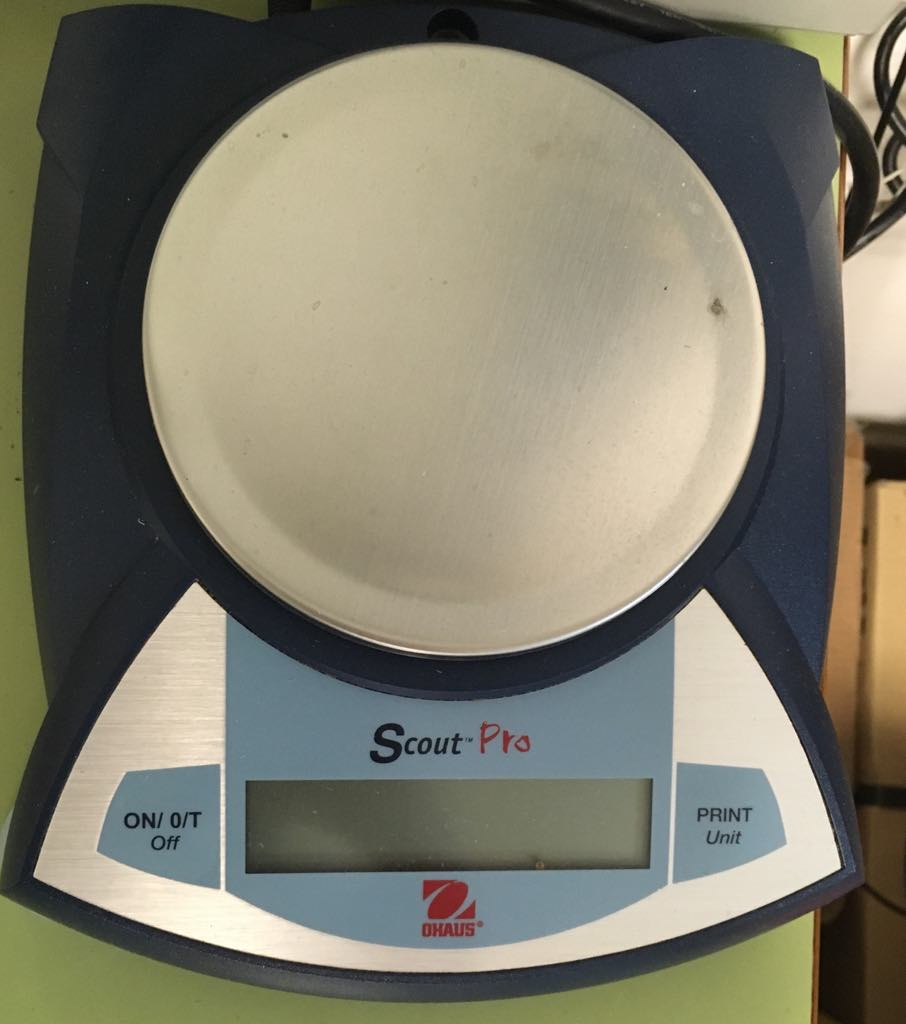

Supplement: S1 Fig — (TIF) [file pone.0234814.s001.tif]

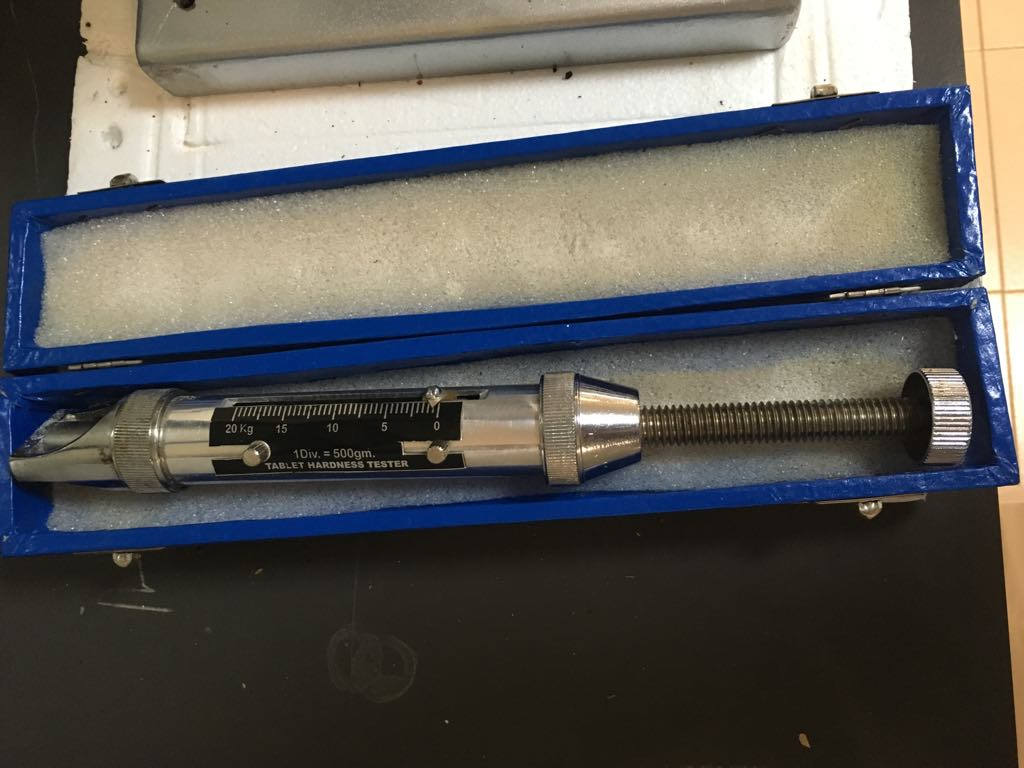

Supplement: S2 Fig — (TIF) [file pone.0234814.s002.tif]

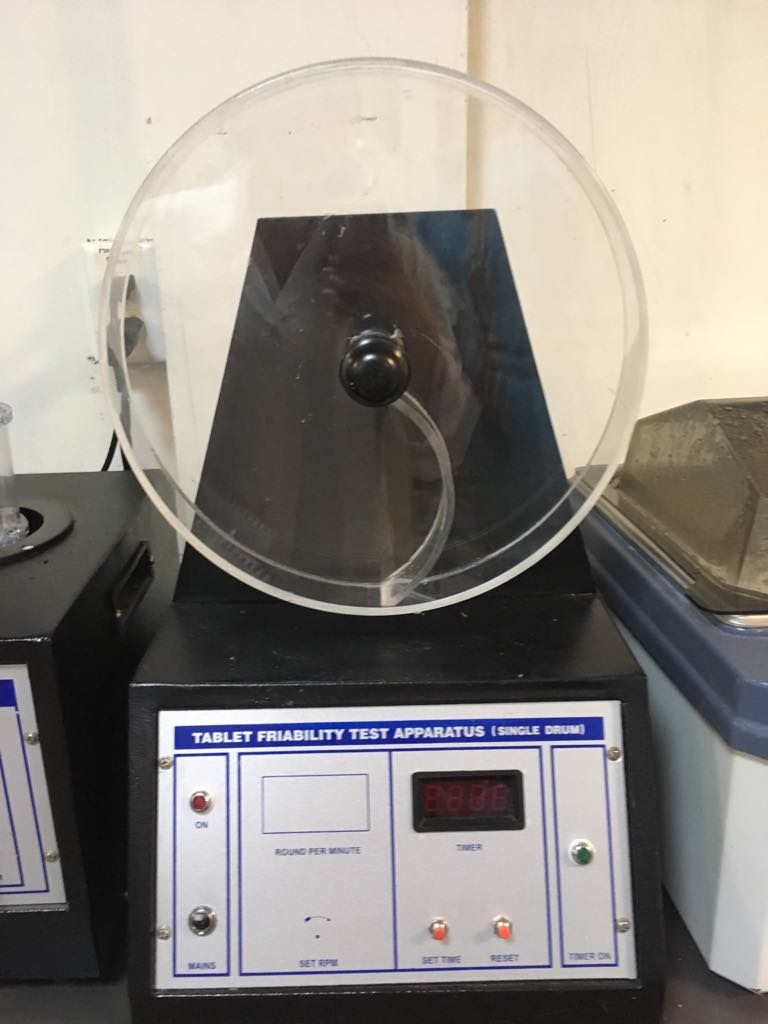

Supplement: S3 Fig — (TIF) [file pone.0234814.s003.tif]

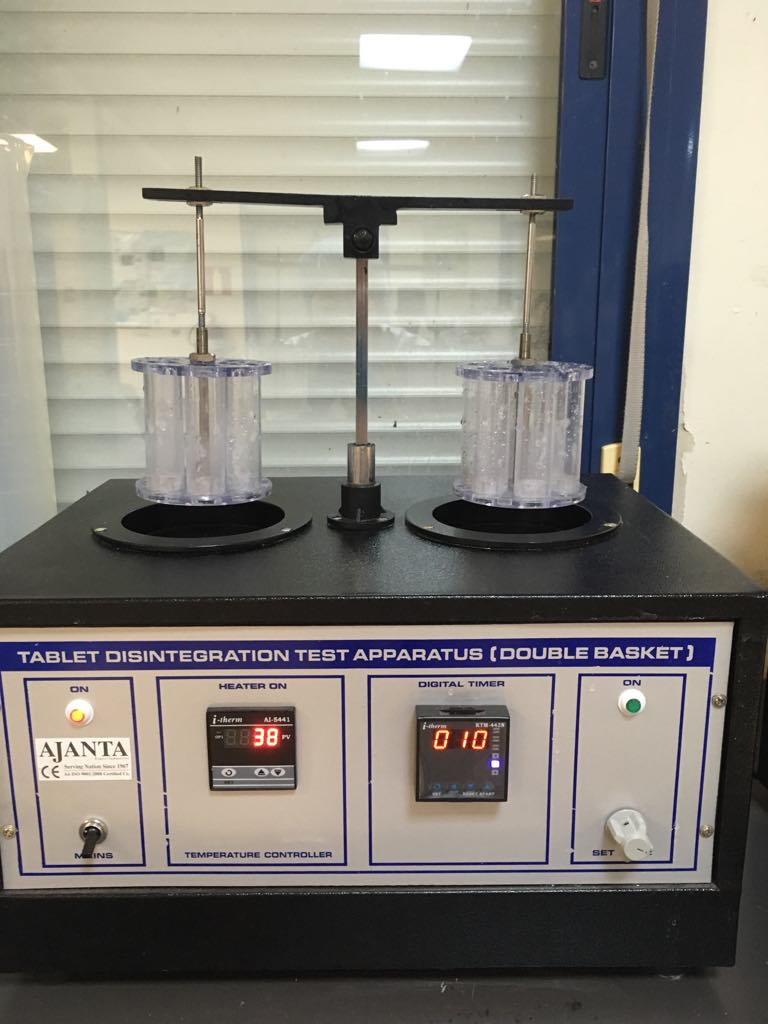

Supplement: S4 Fig — (TIF) [file pone.0234814.s004.tif]
