# Supplementary material for: Field-based screening of selected oral antibiotics in Belize
Source: PLoS One. 2020 Jun 17;15(6):e0234814. doi: 10.1371/journal.pone.0234814 (PMC7299385; doi:10.1371/journal.pone.0234814)
Supplement: S1 Appendix — (PDF) [file pone.0234814.s005.pdf]

Visual Inspection Checklist:

Adapted and Modified from FIP Tool for Visual Inspection of Medicine

1. Packaging

	Yes	No	Other Observations
**1.1 Container and Closure**			
Does the container and closure protect the product from the outside environment; e.g.is the container properly sealed?			
Do they assure that the product will meet the proper specifications throughout its shelf life?			
Are the container and the closure appropriate for the product inside?			
Is the container safely sealed?			

**1.2 Label**			
If there is a carton protecting the container, does the label on the carton match the label on the container?			
Is all information on the label legible and indelible?			

**1.2.1 The trade (brand) name**	Yes	No	Other observations
Is the trade name spelled correctly?			
Is the medicinal product (trade name) registered in the country by the Drug Regulatory Authority)? Is the product legally sold in the country?			
Does the symbol ® follow the trade name?			
For blister or foil strip packed products, is the trade name indelibly impressed or imprinted onto the strip?			
**1.2.2 The active ingredient name (scientific name/generic name):**			

Is the active ingredient name spelt correctly?			
Do the trade name and the active ingredient names correspond to the registered product?			
**1.2.3 The manufacturer's name and logo:**			
Are the manufacturer's name and logo legible and correct?			
Does the logo or hologram (if applicable) look authentic?			
Does the logo or hologram (if applicable) change colour when viewed from different angles?			
**1.2.4 The manufacturer's full address**:			
Is the manufacturer's full address legible and correct?			
Has this company or its agent registered the product in the country?			
**1.2.5 The medicine strength (mg/unit):**			
Is the strength - the amount of active ingredient per unit - clearly stated on the label?			
For blister or foil strip packed products, is the medicine strength indelibly impressed or imprinted onto the strip?			
**1.2.6 The dosage form (e.g., tablet/capsule):**			
Is the dosage form clearly indicated on the container label?			
Does the dosage form stated on the label match the actual dosage form of the medication?			
Is the indicated medicine under this dosage form registered and authorized for sale in the country?			
**1.2.7 The number of units per container:**			
Does the number of dosage units listed on the label match the number of dosage units stated on the			

container?			

**1.2.8 Dosage statement (if appropriate)**	Yes	No	Other observations
Is the dosage clearly indicated on the label?			
Is the dosage stated on the label appropriate for the medicine in this form and strength?			
Is the product registered and authorized for sale in the country with this dosage?			
**1.2.9 The batch (or lot) number:**			
Does the numbering system on the package correspond to that of the producing company?			
For blister or foil strip packed medicines, is the batch number indelibly impressed or imprinted onto the strip?			

2. Physical characteristics of Tablets/Capsules

**2.1 Uniformity of Shape:**	Yes	No	Other observations
Are the tablets/capsules uniform in shape?			
**2.2 Uniformity of Size:**			
Are the tablets/capsules uniform in size?			
**2.3 Uniformity of Colour:**			
Are the tablets/capsules uniform in colour?			
**2.4 Uniformity of Texture:**			
Do the tablets have a uniform coating?			
Is the base of the tablets fully covered?			
Are the tablets uniformly polished, free of powder, and non-sticking?			
**2.5 Markings (scoring, letters, etc):**			
Are markings uniform and identical?			
Does the logo (if present) match that of the			

manufacturing company?			
**2.6 Breaks, Cracks and Splits:**			
Are the tablets/capsules free of breaks, cracks, splits or pinholes?			
**2.7 Embedded surface spots or contamination:**			
Are the tablets/capsules free of embedded surface spots and foreign particle contamination?			
**2.8 Presence of empty capsules in the case of a sample of capsules:**			
Is the sample examined free of empty capsules?			
**2.9 Smell**			
Does the medicine smell the same as the original (If available)? Does it smell peculiar?			
