# Supplementary material for: Field-based screening of selected oral antibiotics in Belize
Source: PLoS One. 2020 Jun 17;15(6):e0234814. doi: 10.1371/journal.pone.0234814 (PMC7299385; doi:10.1371/journal.pone.0234814)
Supplement: S1 Table — (DOCX) [file pone.0234814.s006.docx]

**S1 Table. Visual inspection summary of different brands of Amoxicillin 500mg capsules.**

|  | AMOX C_1_ | AMOX C_2_ | AMOX C_3_ | AMOX C_4_ | AMOX C_5_ |
| --- | --- | --- | --- | --- | --- |
| 1.1 Package container/closure | ALU & transparent PVC | Loose Tablets | ALU & non-transparent white PVC | ALU & non-transparent white PVC | ALU & transparent PVC |
| 1.2 Label | Yes | Yes | Yes | Yes | Yes |
| 1.2.1 The trade (brand) name | Generic | Yes | Generic | Generic | Generic |
| Symbol ® | N/A | No | N/A | N/A | N/A |
| 1.2.2 The active ingredient name | Spanish | English | English | English | English & Spanish |
| 1.2.3 The manufacturer's name and logo | Yes | Yes | Yes | Yes | Yes |
| 1.2.4 The manufacturer's full address | No | Yes | Yes | Yes | No |
| 1.2.5 The medicine strength (mg/unit) |  |  |  |  |  |
| Strength on label | Yes | Yes | Yes | Yes | Yes |
| Medicine strength indelibly impressed or imprinted onto blister/foil | Fades | Loose  Tablets | Yes | Yes | Yes |
| 1.2.6 The dosage form (capsule): | Yes | Yes | Yes | Yes | Yes |
| 1.2.7 The number of units per container | Yes | N/A | Yes | Yes | Yes |
| 1.2.8 Dosage statement (if appropriate) | Rx only | Yes | Rx only | Rx only | Rx only |
| 1.2.9 The batch (or lot) number | Yes | N/A | Yes | Yes | Yes |
| 2.1 Uniformity of Shape: | Yes | Yes | Yes | Yes | Yes |
| 2.2 Uniformity of Size | Yes | Yes | Yes | Yes | Yes |
| 2.3 Uniformity of Color: | Yes | Yes | Yes | Yes | Yes |
| 2.4 Uniformity of Texture (free of powder) | Minimal | Yes | Yes | Minimal | Yes |
| 2.5 Markings (scoring, letters, etc.): | No  Embossing | Yes | Yes | Yes | Yes |
| 2.6 Breaks, Cracks and Splits: | No | No | No | No | No |
| 2.8 Presence of empty capsules | No | No | No | No | No |
| 2.9 Smell with original (if available) | N/A | N/A | N/A | N/A | N/A |
| *Note.* N/A = Not available, ALU = aluminum, PVC = polyvinyl chloride. | | | | | |
