# Supplementary material for: Field-based screening of selected oral antibiotics in Belize
Source: PLoS One. 2020 Jun 17;15(6):e0234814. doi: 10.1371/journal.pone.0234814 (PMC7299385; doi:10.1371/journal.pone.0234814)
Supplement: S2 Table — (DOCX) [file pone.0234814.s007.docx]

**S2 Table. Visual inspection summary of different brands of Co-Trimoxazole 960mg tablets.**

|  | CO-TRI T_1_ | CO-TRI T_2_ | CO-TRI T_3_ | CO-TRI T_4_ | CO-TRI T_5_ |
| --- | --- | --- | --- | --- | --- |
| 1.1 Package container/closure | ALU & transparent PVC | Loose  Tablets | ALU & transparent PVC | Loose  Tablets | ALU & semi-transparent yellow PVC |
| 1.2 Label | Yes | Yes | Yes | Yes | Yes |
| 1.2.1 The trade (brand) name | Generic | Generic | Generic | Yes | Generic |
| Symbol ® | Generic | Generic | Generic | No | Generic |
| 1.2.2 The active ingredient name | Spanish | English | English & Spanish | English | Spanish |
| 1.2.3 The manufacturer's name and logo | Yes | Yes | Yes | Yes | Yes |
| 1.2.4 The manufacturer's full address | Yes | Yes | No | No | No |
| 1.2.5 The medicine strength (mg/unit) |  |  |  |  |  |
| Strength on label | Yes | Yes | Yes | Yes | Yes |
| Information indelibly impressed or imprinted onto blister/foil | Yes | Loose  tablets | Yes | Loose  tablets | Fades |
| 1.2.6 The dosage form (tablet) | Yes | Yes | Yes | Yes | Yes |
| 1.2.7 The number of units per container | Yes | Yes | Yes | Yes | Yes |
| 1.2.8 Dosage statement (if appropriate) | Rx only | No | Rx only | Yes | Rx only |
| 1.2.9 The batch (or lot) number | Yes | Loose  tablets | Yes | Loose  tablets | Yes |
| 2.1 Uniformity of Shape: | Yes | Yes | Yes | Yes | Yes |
| 2.2 Uniformity of Size | Yes | Yes | Yes | Yes | Yes |
| 2.3 Uniformity of Color | Yes | Yes | Yes | Yes | Yes |
| 2.4 Uniformity of Texture |  |  |  |  |  |
| Uniform coating | Yes | Yes | Yes | Yes | Yes |
| Base of the tablets fully covered | Yes | Yes | Yes | Yes | Yes |
| Uniformly polished, free of powder, and non-sticking | Minimal | Minimal | Minimal | Minimal | Evident fine powder |
| 2.5 Markings (scoring, letters, etc.) | Scored | Scored | Scored | Scored | Scored |
| 2.6 Breaks, Cracks and Splits | Slight chipping | None | None | None | Slight chipping |
| 2.7 Embedded surface spots or contamination: | None | None | None | None | None |
| 2.9 Smell | N/A | N/A | N/A | N/A | N/A |
| *Note*. N/A = Not available, ALU = aluminum, PVC = polyvinyl chloride. | | | | | |
