# Supplementary material for: Field-based screening of selected oral antibiotics in Belize
Source: PLoS One. 2020 Jun 17;15(6):e0234814. doi: 10.1371/journal.pone.0234814 (PMC7299385; doi:10.1371/journal.pone.0234814)
Supplement: S3 Table — (DOCX) [file pone.0234814.s008.docx]

**S3 Table. Visual inspection summary of different brands of Ciprofloxacin 500mg tablets.**

|  | CIPRO T_1_ | CIPRO T_2_ | CIPRO T_3_ | CIPRO T_4_ | CIPRO T_5_ |
| --- | --- | --- | --- | --- | --- |
| 1.1 Package container/closure | ALU-ALU | ALU & transparent PVC | ALU & transparent PVC | ALU & semi-transparent brown PVC | ALU & transparent PVC |
| 1.2 Label legible | YES | YES | YES | YES | YES |
| 1.2.1 The trade (brand) name | YES | GENERIC | GENERIC | GENERIC | GENERIC |
| Symbol ® | NO | N/A | N/A | N/A | N/A |
| 1.2.2 The active ingredient name: | English | Spanish | Spanish | English & Spanish | English |
| 1.2.3 The manufacturer's name and logo: | YES | YES | YES | YES | YES |
| 1.2.4 The manufacturer's full address: | NO | YES | NO | NO | NO |
| 1.2.5 The medicine strength (mg/unit): |  |  |  |  |  |
| Strength on label | YES | YES | YES | YES | YES |
| Medicine strength indelibly impressed or imprinted onto blister/foil | YES | YES | FADES | YES | YES |
| 1.2.6 The dosage form (tablet): | YES | YES | YES | YES | YES |
| 1.2.7 The number of units per container: | YES | YES | YES | YES | YES |
| 1.2.8 Dosage statement (if appropriate) | PHYSICIAN | Rx only | Rx only | PHYSICIAN | DOCTOR |
| 1.2.9 The batch (or lot) number: | YES | YES | YES | YES | YES |
| 2.1 Uniformity of Shape: | YES | YES | YES | YES | YES |
| 2.2 Uniformity of Size: | YES | YES | YES | YES | YES |
| 2.3 Uniformity of Color: | YES | YES | NO | YES | YES |
| 2.4 Uniformity of Texture: |  |  |  |  |  |
| Uniform coating | YES | YES | NO | YES | YES |
| Base of the tablets fully covered | YES | YES | NO | YES | YES |
| Uniformly polished, free of powder, and non-sticking | POWDERED | SLIGHT POWDER | SLIGHT POWDER | MINIMAL POWDER | NONE |
| 2.5 Markings (scoring, letters, etc.): | UNEVEN SCORING | NO SCORING | SCORED | SCORED | SCCORED AND CODED |
| 2.6 Breaks, Cracks and Splits: | SLIGHT CHIPPING | ROUGH SURFACE | SLIGHT CHIPPING | NONE | NONE |
| 2.7 Embedded surface spots or contamination: | NONE | NONE | NONE | NONE | NONE |
| 2.9 Smell | N/A | N/A | N/A | N/A | N/A |
| N/A = Not available, ALU = aluminum, PVC = polyvinyl chloride. | | | | | |
