# Supplementary material for: Field-based screening of selected oral antibiotics in Belize
Source: PLoS One. 2020 Jun 17;15(6):e0234814. doi: 10.1371/journal.pone.0234814 (PMC7299385; doi:10.1371/journal.pone.0234814)
Supplement: S4 Table — (DOCX) [file pone.0234814.s009.docx]

**S4 Table. Weight uniformity of USP Amoxicillin 500mg capsules.**

|  | AMOX C_1_(gm) | | | AMOX C_2_(gm) | | |
| --- | --- | --- | --- | --- | --- | --- |
|  | **Whole** | **Powder** | **Shell** | **Whole** | **Powder** | **Shell** |
| 1 | 0.67 | 0.58 | 0.09 | 0.69 | 0.59 | 0.10 |
| 2 | 0.68 | 0.59 | 0.09 | 0.72 | 0.62 | 0.10 |
| 3 | 0.69 | 0.59 | 0.10 | 0.71 | 0.61 | 0.10 |
| 4 | 0.69 | 0.60 | 0.09 | 0.72 | 0.62 | 0.10 |
| 5 | 0.71 | 0.62 | 0.09 | 0.72 | 0.62 | 0.10 |
| 6 | 0.71 | 0.63 | 0.08 | 0.71 | 0.61 | 0.10 |
| 7 | 0.70 | 0.62 | 0.08 | 0.72 | 0.62 | 0.10 |
| 8 | 0.65 | 0.55 | 0.10 | 0.72 | 0.62 | 0.10 |
| 9 | 0.72 | 0.62 | 0.10 | 0.72 | 0.62 | 0.10 |
| 10 | 0.68 | 0.59 | 0.09 | 0.72 | 0.62 | 0.10 |
| 11 | 0.66 | 0.57 | 0.09 | 0.72 | 0.62 | 0.10 |
| 12 | 0.70 | 0.61 | 0.09 | 0.72 | 0.62 | 0.10 |
| 13 | 0.72 | 0.62 | 0.10 | 0.72 | 0.62 | 0.10 |
| 14 | 0.72 | 0.63 | 0.09 | 0.73 | 0.63 | 0.10 |
| 15 | 0.69 | 0.60 | 0.09 | 0.72 | 0.62 | 0.10 |
| 16 | 0.73 | 0.64 | 0.09 | 0.71 | 0.61 | 0.10 |
| 17 | 0.65 | 0.56 | 0.09 | 0.73 | 0.63 | 0.10 |
| 18 | 0.69 | 0.59 | 0.10 | 0.72 | 0.62 | 0.10 |
| 19 | 0.70 | 0.61 | 0.09 | 0.73 | 0.63 | 0.10 |
| 20 | 0.59 | 0.50 | 0.09 | 0.72 | 0.62 | 0.10 |
| 21 | 0.68 | 0.59 | 0.09 | 0.71 | 0.61 | 0.10 |
| 22 | 0.65 | 0.55 | 0.10 | 0.73 | 0.63 | 0.10 |
| 23 | 0.69 | 0.60 | 0.09 | 0.71 | 0.61 | 0.10 |
| 24 | 0.70 | 0.61 | 0.09 | 0.72 | 0.62 | 0.10 |
| 25 | 0.69 | 0.60 | 0.09 | 0.72 | 0.62 | 0.10 |
| 26 | 0.70 | 0.61 | 0.09 | 0.72 | 0.62 | 0.10 |
| 27 | 0.70 | 0.61 | 0.09 | 0.73 | 0.63 | 0.10 |
| 28 | 0.70 | 0.60 | 0.10 | 0.71 | 0.61 | 0.10 |
| 29 | 0.67 | 0.58 | 0.09 | 0.72 | 0.62 | 0.10 |
| 30 | 0.71 | 0.61 | 0.10 | 0.71 | 0.61 | 0.10 |
| MEAN | **0.69** | **0.60** | **0.09** | **0.72** | **0.62** | **0.10** |
| SD | **0.0281** | **0.0287** | **0.0055** | **0.0083** | **0.0083** | **0.0000** |
|  | | | | |  |  |
