# Supplementary material for: Field-based screening of selected oral antibiotics in Belize
Source: PLoS One. 2020 Jun 17;15(6):e0234814. doi: 10.1371/journal.pone.0234814 (PMC7299385; doi:10.1371/journal.pone.0234814)
Supplement: S5 Table — (DOCX) [file pone.0234814.s010.docx]

**S5 Table. Weight uniformity of BP Amoxicillin 500mg capsules.**

|  | AMOX C_3_ (g) | | | AMOX C_4_ (g) | | | AMOX C_5_ (g) | | |
| --- | --- | --- | --- | --- | --- | --- | --- | --- | --- |
|  | **Whole** | **Powder** | **Shell** | **Whole** | **Powder** | **Shell** | **Whole** | **Powder** | **Shell** |
| 1 | 0.67 | 0.57 | 0.10 | 0.68 | 0.58 | 0.10 | 0.70 | 0.59 | 0.11 |
| 2 | 0.66 | 0.56 | 0.10 | 0.67 | 0.58 | 0.09 | 0.71 | 0.60 | 0.11 |
| 3 | 0.67 | 0.57 | 0.10 | 0.63 | 0.54 | 0.09 | 0.69 | 0.58 | 0.11 |
| 4 | 0.66 | 0.57 | 0.09 | 0.68 | 0.59 | 0.09 | 0.70 | 0.60 | 0.10 |
| 5 | 0.67 | 0.58 | 0.09 | 0.70 | 0.61 | 0.09 | 0.72 | 0.61 | 0.11 |
| 6 | 0.68 | 0.58 | 0.10 | 0.68 | 0.58 | 0.10 | 0.69 | 0.58 | 0.11 |
| 7 | 0.68 | 0.58 | 0.10 | 0.69 | 0.60 | 0.09 | 0.70 | 0.59 | 0.11 |
| 8 | 0.66 | 0.56 | 0.10 | 0.67 | 0.58 | 0.09 | 0.70 | 0.59 | 0.11 |
| 9 | 0.66 | 0.56 | 0.10 | 0.69 | 0.59 | 0.10 | 0.69 | 0.58 | 0.11 |
| 10 | 0.66 | 0.57 | 0.09 | 0.68 | 0.59 | 0.09 | 0.70 | 0.59 | 0.11 |
| 11 | 0.66 | 0.56 | 0.10 | 0.65 | 0.56 | 0.09 | 0.73 | 0.62 | 0.11 |
| 12 | 0.66 | 0.57 | 0.09 | 0.68 | 0.59 | 0.09 | 0.71 | 0.61 | 0.10 |
| 13 | 0.67 | 0.58 | 0.09 | 0.64 | 0.55 | 0.09 | 0.72 | 0.60 | 0.12 |
| 14 | 0.66 | 0.57 | 0.09 | 0.71 | 0.62 | 0.09 | 0.71 | 0.60 | 0.11 |
| 15 | 0.67 | 0.57 | 0.10 | 0.68 | 0.59 | 0.09 | 0.69 | 0.58 | 0.11 |
| 16 | 0.66 | 0.56 | 0.10 | 0.67 | 0.58 | 0.09 | 0.70 | 0.60 | 0.10 |
| 17 | 0.68 | 0.58 | 0.10 | 0.68 | 0.59 | 0.09 | 0.69 | 0.58 | 0.11 |
| 18 | 0.67 | 0.57 | 0.10 | 0.69 | 0.60 | 0.09 | 0.70 | 0.59 | 0.11 |
| 19 | 0.69 | 0.59 | 0.10 | 0.66 | 0.57 | 0.09 | 0.68 | 0.58 | 0.10 |
| 20 | 0.66 | 0.56 | 0.10 | 0.68 | 0.59 | 0.09 | 0.68 | 0.57 | 0.11 |
| 21 | 0.68 | 0.59 | 0.09 | 0.63 | 0.53 | 0.10 | 0.69 | 0.57 | 0.12 |
| 22 | 0.67 | 0.57 | 0.10 | 0.68 | 0.59 | 0.09 | 0.71 | 0.60 | 0.11 |
| 23 | 0.65 | 0.55 | 0.10 | 0.65 | 0.55 | 0.10 | 0.68 | 0.58 | 0.10 |
| 24 | 0.67 | 0.57 | 0.10 | 0.69 | 0.60 | 0.09 | 0.69 | 0.58 | 0.11 |
| 25 | 0.66 | 0.56 | 0.10 | 0.67 | 0.57 | 0.10 | 0.68 | 0.58 | 0.10 |
| 26 | 0.68 | 0.58 | 0.10 | 0.68 | 0.59 | 0.09 | 0.69 | 0.58 | 0.11 |
| 27 | 0.67 | 0.58 | 0.09 | 0.66 | 0.57 | 0.09 | 0.69 | 0.58 | 0.11 |
| 28 | 0.65 | 0.55 | 0.10 | 0.67 | 0.57 | 0.10 | 0.71 | 0.60 | 0.11 |
| 29 | 0.66 | 0.56 | 0.10 | 0.70 | 0.61 | 0.09 | 0.68 | 0.58 | 0.10 |
| 30 | 0.66 | 0.57 | 0.09 | 0.66 | 0.56 | 0.10 | 0.70 | 0.59 | 0.11 |
| MEAN | **0.67** | **0.57** | **0.10** | **0.67** | **0.58** | **0.09** | **0.70** | **0.59** | **0.11** |
| SD | **0.0096** | **0.0103** | **0.0047** | **0.0194** | **0.0208** | **0.0045** | **0.0130** | **0.0123** | **0.0053** |
| W = whole capsules, P = powder, S = shell | | | | | | | | | |
