# Supplementary material for: Field-based screening of selected oral antibiotics in Belize
Source: PLoS One. 2020 Jun 17;15(6):e0234814. doi: 10.1371/journal.pone.0234814 (PMC7299385; doi:10.1371/journal.pone.0234814)
Supplement: S6 Table — (DOCX) [file pone.0234814.s011.docx]

**S6 Table. Weight uniformity of Co-Trimoxazole 960mg.**

|  | BP | | | USP | |
| --- | --- | --- | --- | --- | --- |
|  | **CO-TRI T_1_**(g) | **CO-TRI T_2_**(g) | **CO-TRI T_3_**(g) | **CO-TRI T_4_**(g) | **CO-TRI T_5_**(g) |
| 1 | 1.03 | 1.17 | 1.03 | 1.19 | 0.99 |
| 2 | 1.03 | 1.20 | 1.03 | 1.19 | 1.03 |
| 3 | 1.03 | 1.20 | 1.04 | 1.19 | 1.02 |
| 4 | 1.01 | 1.20 | 1.03 | 1.19 | 1.05 |
| 5 | 1.00 | 1.20 | 1.03 | 1.18 | 1.00 |
| 6 | 1.01 | 1.20 | 1.04 | 1.19 | 1.00 |
| 7 | 1.03 | 1.19 | 1.03 | 1.19 | 1.06 |
| 8 | 1.02 | 1.19 | 1.03 | 1.20 | 1.03 |
| 9 | 1.01 | 1.20 | 1.03 | 1.20 | 1.03 |
| 10 | 1.02 | 1.20 | 1.03 | 1.22 | 1.02 |
| 11 | 1.01 | 1.19 | 1.03 | 1.19 | 1.00 |
| 12 | 1.03 | 1.20 | 1.03 | 1.19 | 1.00 |
| 13 | 1.00 | 1.21 | 1.03 | 1.19 | 1.03 |
| 14 | 1.01 | 1.15 | 1.03 | 1.18 | 0.99 |
| 15 | 1.01 | 1.20 | 1.04 | 1.18 | 1.03 |
| 16 | 1.03 | 1.21 | 1.03 | 1.20 | 1.03 |
| 17 | 1.04 | 1.19 | 1.03 | 1.19 | 1.02 |
| 18 | 1.02 | 1.19 | 1.03 | 1.20 | 1.00 |
| 19 | 1.02 | 1.20 | 1.04 | 1.20 | 0.98 |
| 20 | 1.00 | 1.20 | 1.04 | 1.19 | 1.03 |
| 21 | 1.03 | 1.19 | 1.03 | 1.20 | 1.01 |
| 22 | 1.01 | 1.20 | 1.03 | 1.18 | 1.02 |
| 23 | 1.01 | 1.20 | 1.03 | 1.18 | 1.02 |
| 24 | 1.02 | 1.18 | 1.03 | 1.19 | 1.01 |
| 25 | 1.01 | 1.19 | 1.03 | 1.22 | 1.00 |
| 26 | 1.05 | 1.21 | 1.04 | 1.20 | 1.02 |
| 27 | 1.03 | 1.20 | 1.03 | 1.19 | 1.04 |
| 28 | 1.04 | 1.20 | 1.04 | 1.20 | 1.01 |
| 29 | 1.04 | 1.20 | 1.03 | 1.19 | 0.99 |
| 30 | 1.02 | 1.21 | 1.04 | 1.20 | 1.04 |
| Mean | **1.02** | **1.20** | **1.03** | **1.19** | **1.02** |
| SD | **0.0131** | **0.0122** | **0.0045** | **0.0099** | **0.0192** |
