# Supplementary material for: Field-based screening of selected oral antibiotics in Belize
Source: PLoS One. 2020 Jun 17;15(6):e0234814. doi: 10.1371/journal.pone.0234814 (PMC7299385; doi:10.1371/journal.pone.0234814)
Supplement: S7 Table — (DOCX) [file pone.0234814.s012.docx]

**S7 Table. Weight uniformity of Ciprofloxacin 500mg.**

|  | **USP** | | | | **BP** |
| --- | --- | --- | --- | --- | --- |
|  | **CIPRO T_1_**(g) | **CIPRO T_2_**(g) | **CIPRO T_3_**(g) | **CIPRO T_4_**(g) | **CIPRO T_5_**(g) |
| **1** | 0.97 | 0.67 | 0.88 | 0.92 | 0.69 |
| **2** | 0.99 | 0.67 | 0.87 | 0.93 | 0.69 |
| **3** | 0.97 | 0.68 | 0.87 | 0.94 | 0.68 |
| **4** | 0.97 | 0.66 | 0.88 | 0.94 | 0.68 |
| **5** | 0.97 | 0.67 | 0.89 | 0.94 | 0.70 |
| **6** | 0.98 | 0.67 | 0.88 | 0.93 | 0.68 |
| **7** | 0.98 | 0.70 | 0.87 | 0.93 | 0.69 |
| **8** | 0.97 | 0.66 | 0.87 | 0.93 | 0.69 |
| **9** | 0.98 | 0.69 | 0.86 | 0.93 | 0.69 |
| **10** | 0.97 | 0.65 | 0.88 | 0.94 | 0.69 |
| **11** | 0.95 | 0.70 | 0.87 | 0.93 | 0.69 |
| **12** | 0.98 | 0.67 | 0.85 | 0.92 | 0.69 |
| **13** | 0.98 | 0.67 | 0.88 | 0.93 | 0.70 |
| **14** | 0.97 | 0.73 | 0.87 | 0.93 | 0.68 |
| **15** | 0.96 | 0.68 | 0.88 | 0.93 | 0.69 |
| **16** | 0.97 | 0.67 | 0.85 | 0.93 | 0.70 |
| **17** | 0.97 | 0.64 | 0.88 | 0.94 | 0.69 |
| **18** | 0.95 | 0.66 | 0.88 | 0.92 | 0.70 |
| **19** | 0.97 | 0.68 | 0.89 | 0.93 | 0.68 |
| **20** | 0.97 | 0.68 | 0.88 | 0.94 | 0.70 |
| **21** | 1.02 | 0.69 | 0.88 | 0.94 | 0.69 |
| **22** | 1.00 | 0.68 | 0.88 | 0.93 | 0.70 |
| **23** | 0.96 | 0.72 | 0.86 | 0.94 | 0.68 |
| **24** | 0.98 | 0.70 | 0.89 | 0.96 | 0.70 |
| **25** | 0.96 | 0.66 | 0.87 | 0.92 | 0.69 |
| **26** | 0.97 | 0.71 | 0.84 | 0.94 | 0.69 |
| **27** | 0.96 | 0.67 | 0.86 | 0.94 | 0.70 |
| **28** | 0.97 | 0.66 | 0.88 | 0.94 | 0.69 |
| **29** | 0.96 | 0.68 | 0.88 | 0.94 | 0.67 |
| **30** | 0.97 | 0.64 | 0.88 | 0.92 | 0.69 |
| **MEAN** | **0.97** | **0.68** | **0.87** | **0.93** | **0.69** |
| **SD** | **0.0138** | **0.0212** | **0.0121** | **0.0088** | **0.0079** |
