# Supplementary material for: Field-based screening of selected oral antibiotics in Belize
Source: PLoS One. 2020 Jun 17;15(6):e0234814. doi: 10.1371/journal.pone.0234814 (PMC7299385; doi:10.1371/journal.pone.0234814)
Supplement: S8 Table — (DOCX) [file pone.0234814.s013.docx]

**S8 Table. Friability test for BP Co-Trimoxazole 960mg tablets.**

|  | CO-TRI T_1_ (mg) | | | CO-TRI T_2_ (min) | | | CO-TRI T_3_ (min) | | |
| --- | --- | --- | --- | --- | --- | --- | --- | --- | --- |
|  | 1 | 2 | 3 | 1 | 2 | 3 | 1 | 2 | 3 |
| Initial | 10.23 | 10.25 | 10.23 | 11.99 | 11.97 | 11.96 | 10.36 | 10.35 | 10.36 |
| Final | 10.16 | 10.11 | 10.16 | 11.97 | 11.95 | 11.93 | 10.32 | 10.32 | 10.32 |
| % Loss | **0.68** | **1.37** | **0.68** | **0.17** | **0.17** | **0.25** | **0.39** | **0.29** | **0.39** |
