# Supplementary material for: Field-based screening of selected oral antibiotics in Belize
Source: PLoS One. 2020 Jun 17;15(6):e0234814. doi: 10.1371/journal.pone.0234814 (PMC7299385; doi:10.1371/journal.pone.0234814)
Supplement: S9 Table — (DOCX) [file pone.0234814.s014.docx]

**S9 Table. Friability test for USP Co-Trimoxazole 960mg tablets.**

|  | CO-TRI T_4_ (mg) | | | CO-TRI T_5_ (mg) | | |
| --- | --- | --- | --- | --- | --- | --- |
|  | 1 | 2 | 3 | 1 | 2 | 3 |
| Initial | 11.95 | 11.99 | 11.96 | 10.11 | 10.11 | 10.25 |
| Final | 11.93 | 11.97 | 11.95 | 10.08 | 10.04 | 10.21 |
| % loss | **0.17** | **0.17** | **0.08** | **0.30** | **0.69** | **0.39** |
