# Supplementary material for: Field-based screening of selected oral antibiotics in Belize
Source: PLoS One. 2020 Jun 17;15(6):e0234814. doi: 10.1371/journal.pone.0234814 (PMC7299385; doi:10.1371/journal.pone.0234814)
Supplement: S10 Table — (DOCX) [file pone.0234814.s015.docx]

**S10 Table. Friability test for USP Ciprofloxacin 500mg tablets.**

|  | **CIPRO T_1_**(mg) | | | **CIPRO T_2_**(mg) | | | **CIPRO T_3_**(mg) | | | **CIPRO T_4_**(mg) | | |
| --- | --- | --- | --- | --- | --- | --- | --- | --- | --- | --- | --- | --- |
|  | 1 | 2 | 3 | 1 | 2 | 3 | 1 | 2 | 3 | 1 | 2 | 3 |
| **INITIAL** | 9.71 | 9.78 | 9.77 | 6.80 | 6.80 | 6.67 | 8.73 | 8.73 | 8.67 | 9.33 | 9.31 | 9.32 |
| **FINAL** | 9.67 | 9.71 | 9.69 | 6.80 | 6.80 | 6.66 | 8.68 | 8.70 | 8.63 | 9.31 | 9.30 | 9.31 |
| **% LOSS** | **0.41** | **0.72** | **0.82** | **0.00** | **0.00** | **0.15** | **0.57** | **0.34** | **0.46** | **0.21** | **0.11** | **0.11** |
