# Supplementary material for: Field-based screening of selected oral antibiotics in Belize
Source: PLoS One. 2020 Jun 17;15(6):e0234814. doi: 10.1371/journal.pone.0234814 (PMC7299385; doi:10.1371/journal.pone.0234814)
Supplement: S11 Table — (DOCX) [file pone.0234814.s016.docx]

**S11 Table. Friability test for BP Ciprofloxacin 500mg tablets.**

|  | **CIPRO T_5_ (**mg**)** | | |
| --- | --- | --- | --- |
|  | 1 | 2 | 3 |
| **INITIALWEIGHT** | 6.95 | 6.97 | 6.96 |
| **FINAL WEIGHT** | 6.94 | 6.97 | 6.96 |
| **% LOSS** | **0.14** | **0.00** | **0.00** |
